# Supplementary material for: Promiscuous Lipase-Catalyzed Knoevenagel–Phospha–Michael Reaction for the Synthesis of Antimicrobial β-Phosphono Malonates
Source: Int J Mol Sci. 2022 Aug 8;23(15):8819. doi: 10.3390/ijms23158819 (PMC9368977; doi:10.3390/ijms23158819)
Supplement: Supplementary file 1 [file ijms-23-08819-s001.zip › ijms-1841688-supplementary.pdf]

Supplementary Materials

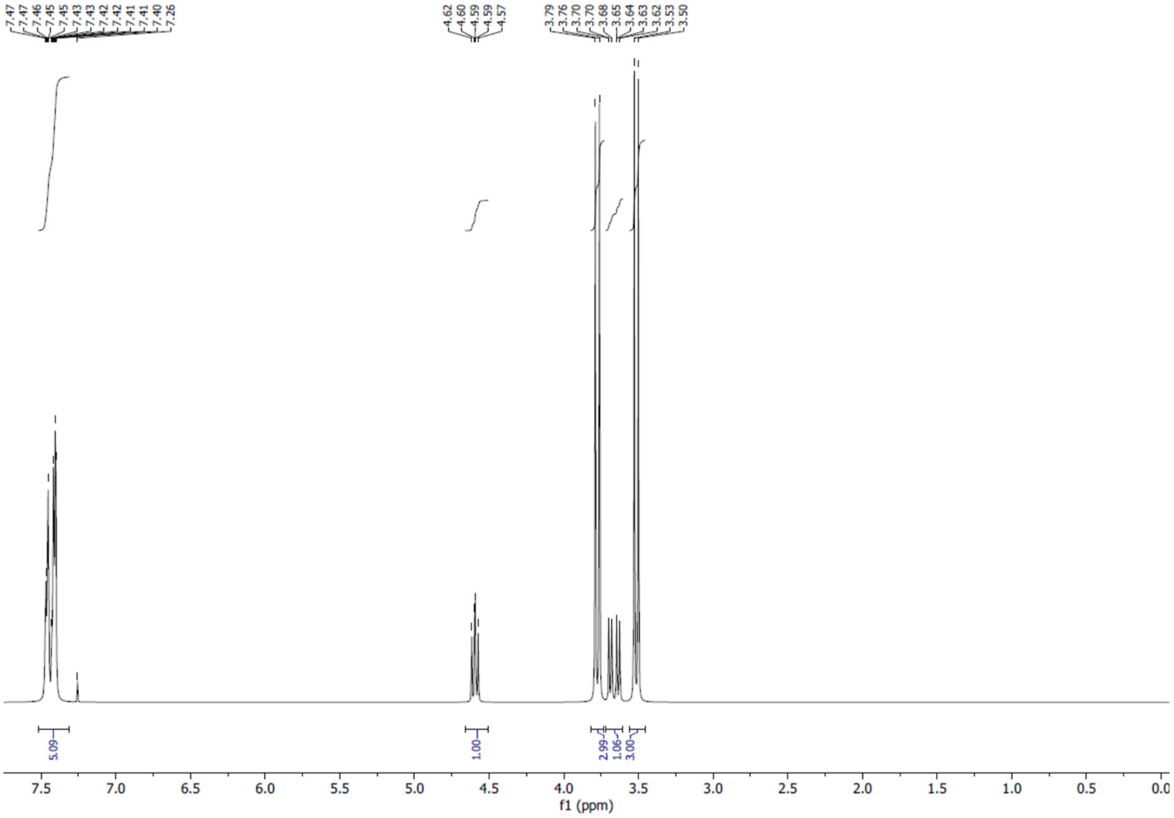

Figure S1. ^{1}HNMR (400 MHz, $CDCl_3$) spectra of dimethyl (2,2-dicyano-1-phenylethyl)phosphonate (**1**).

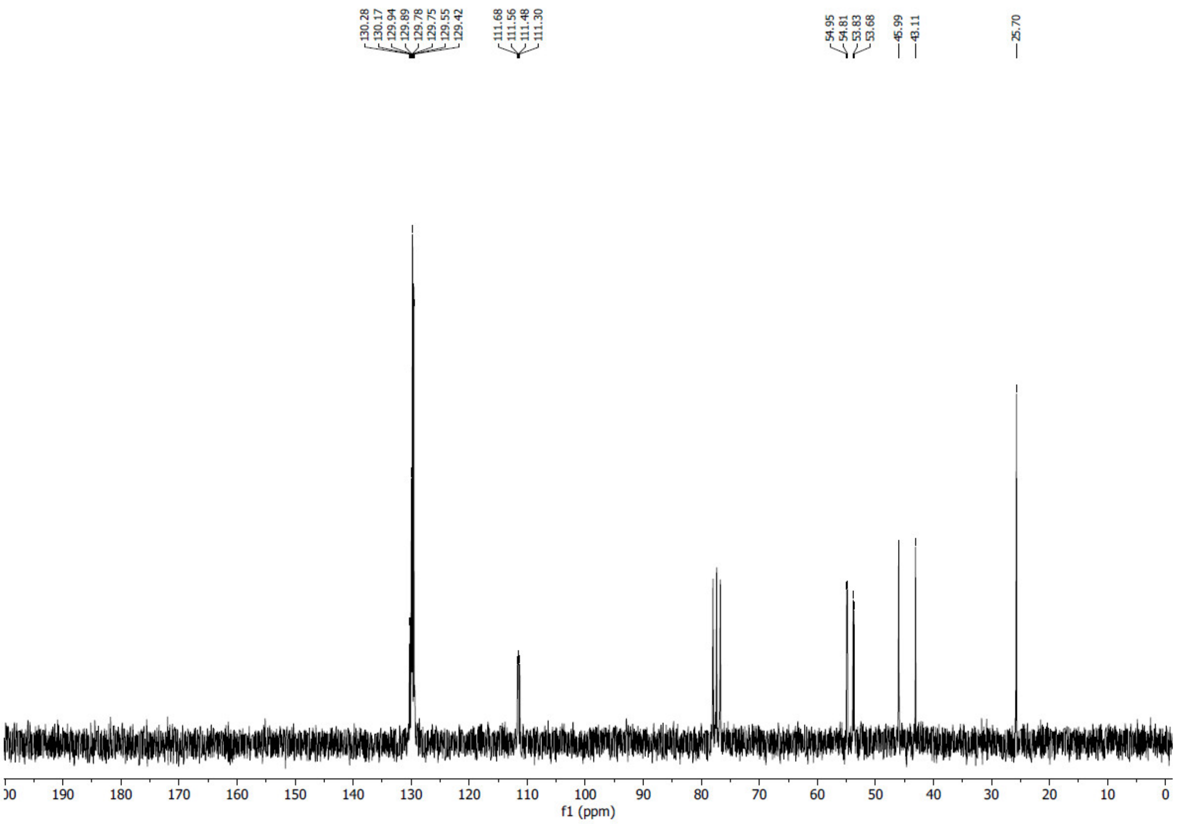

Figure S2. ^{13}CNMR (100 MHz, $CDCl_3$) spectra of dimethyl (2,2-dicyano-1-phenylethyl)phosphonate (**1**).

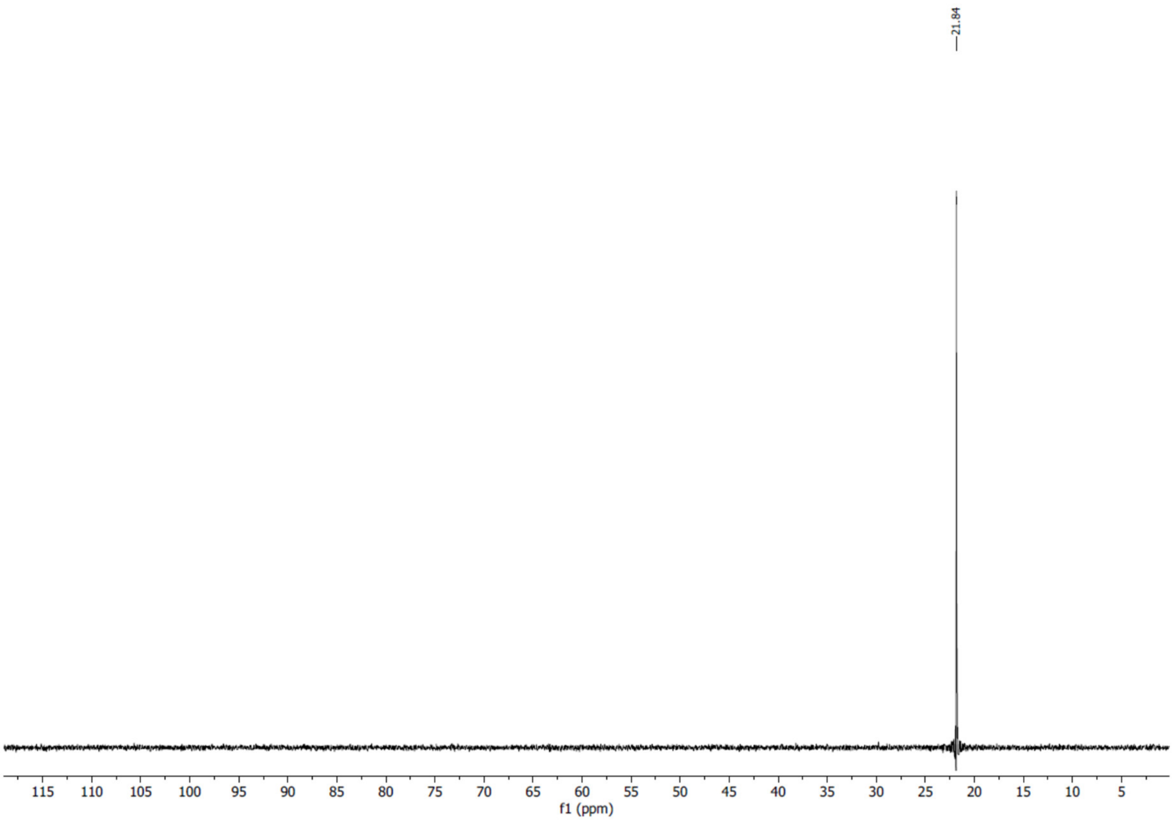

Figure S3. ^{31}PNMR (162 MHz, $CDCl_3$) spectra of dimethyl (2,2-dicyano-1-phenylethyl)phosphonate (**1**).

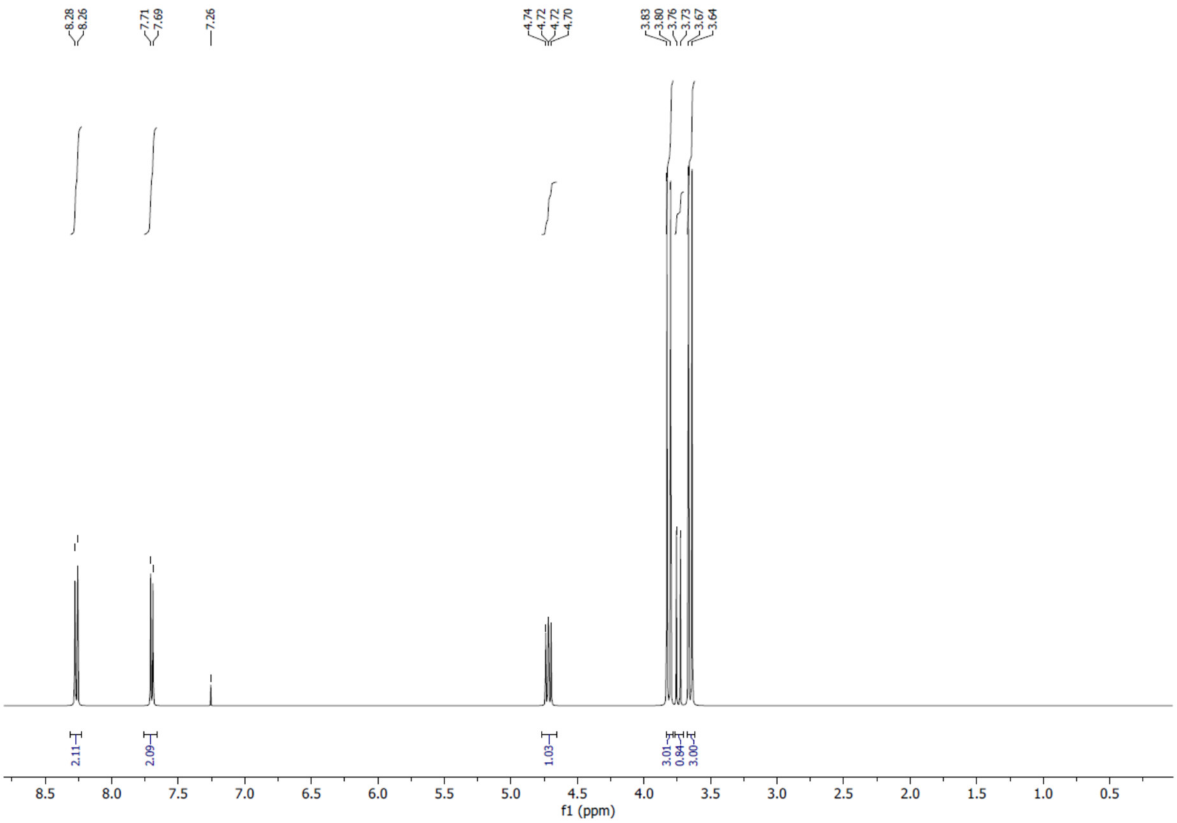

Figure S4. ^{1}HNMR (400 MHz, $CDCl_3$) spectra of dimethyl (2,2-dicyano-1-(4-methoxyphenyl)ethyl)phosphonate (**2**).

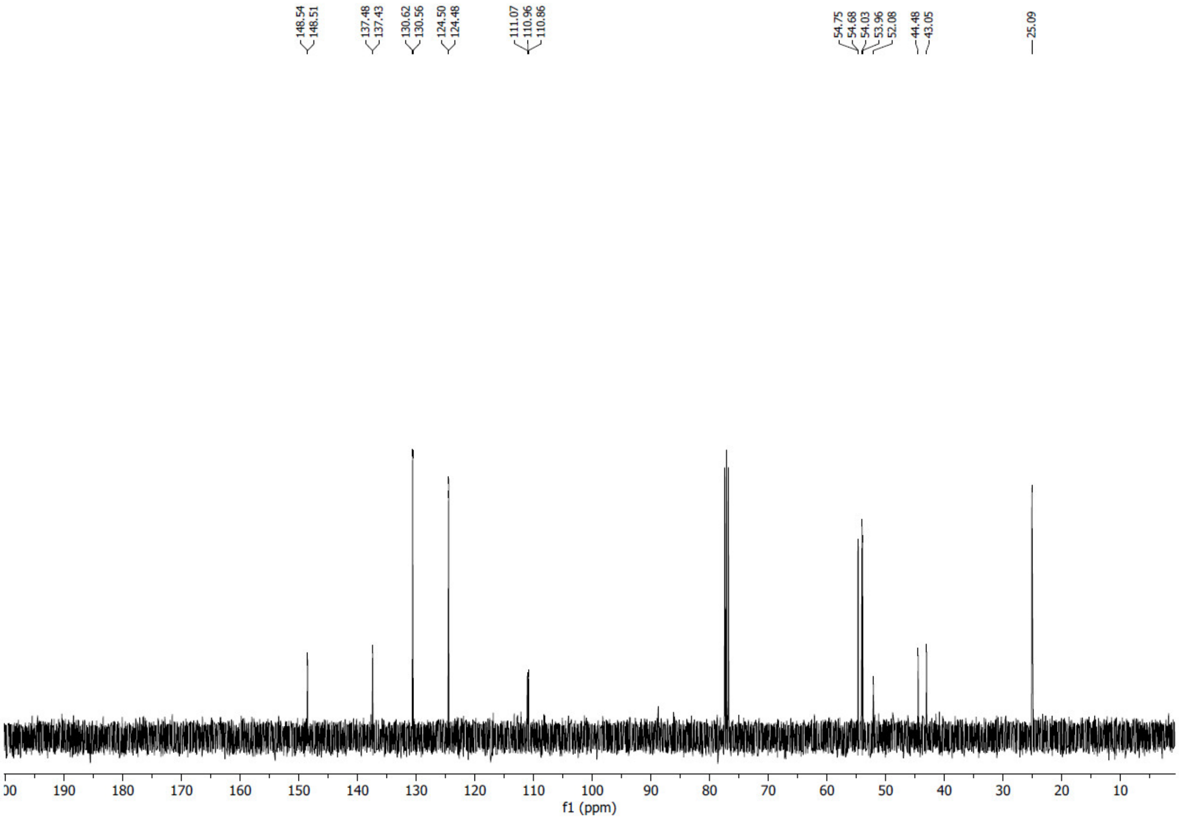

Figure S5. ^{13}CNMR (100 MHz, $CDCl_3$) spectra of dimethyl (2,2-dicyano-1-(4-methoxyphenyl)ethyl)phosphonate (**2**).

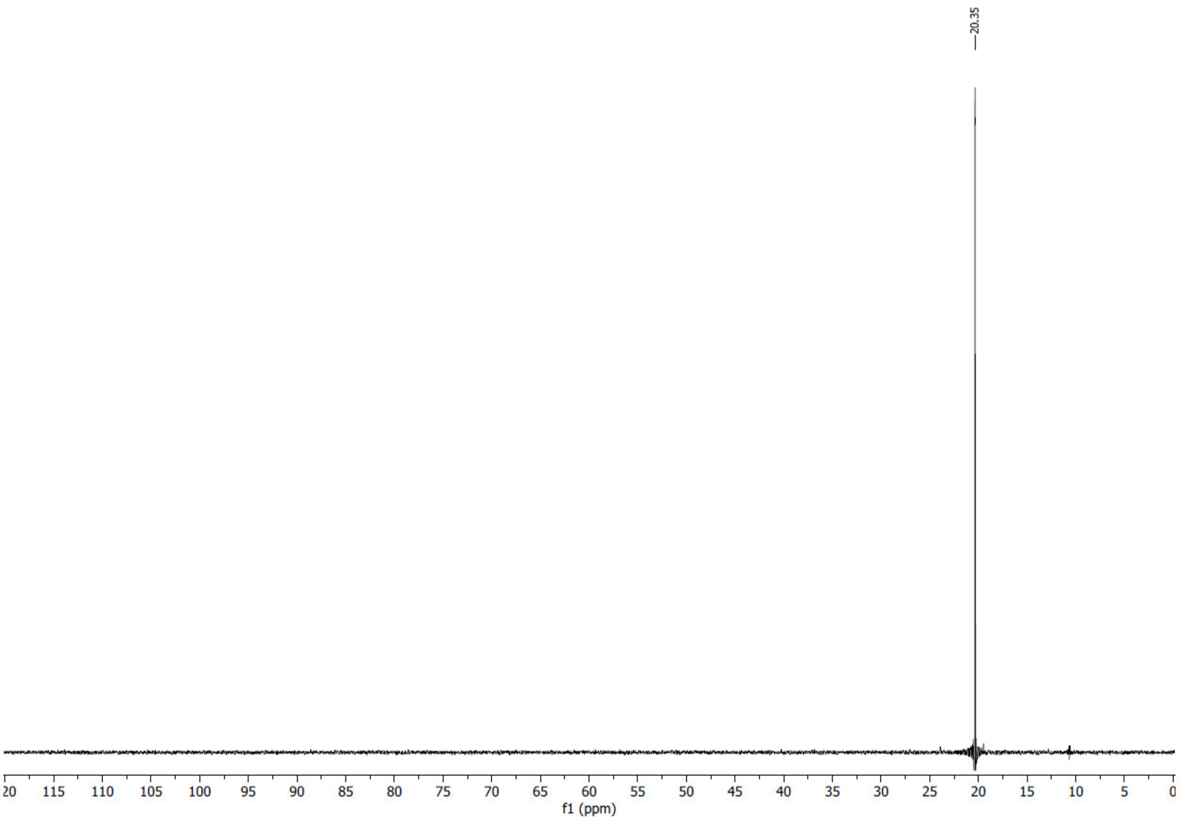

Figure S6. 31PNMR (162 MHz, $CDCl_3$) spectra of dimethyl (2,2-dicyano-1-(4-methoxyphenyl)ethyl)phosphonate (**2**).

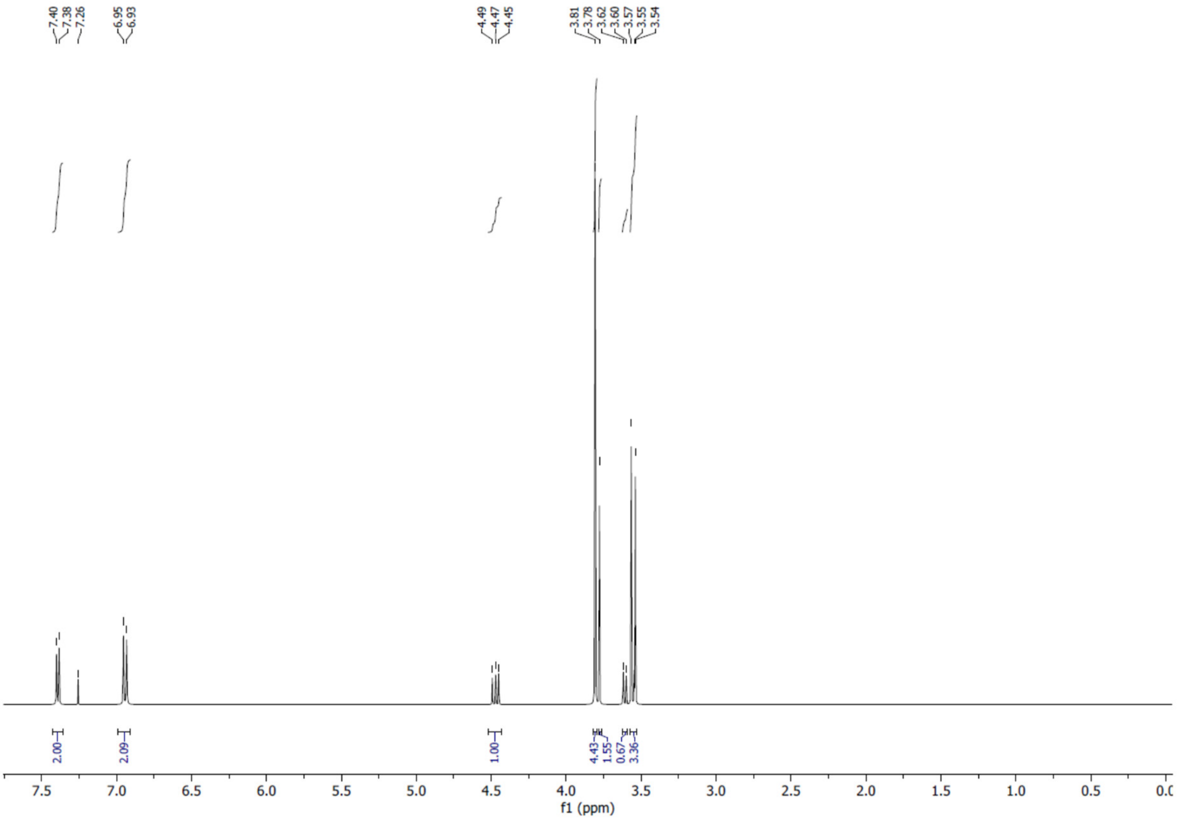

Figure S7. 1NNMR (400 MHz, $CDCl_3$) spectra of dimethyl (2,2-dicyano-1-(4-methoxyphenyl)ethyl)phosphonate (**3**)

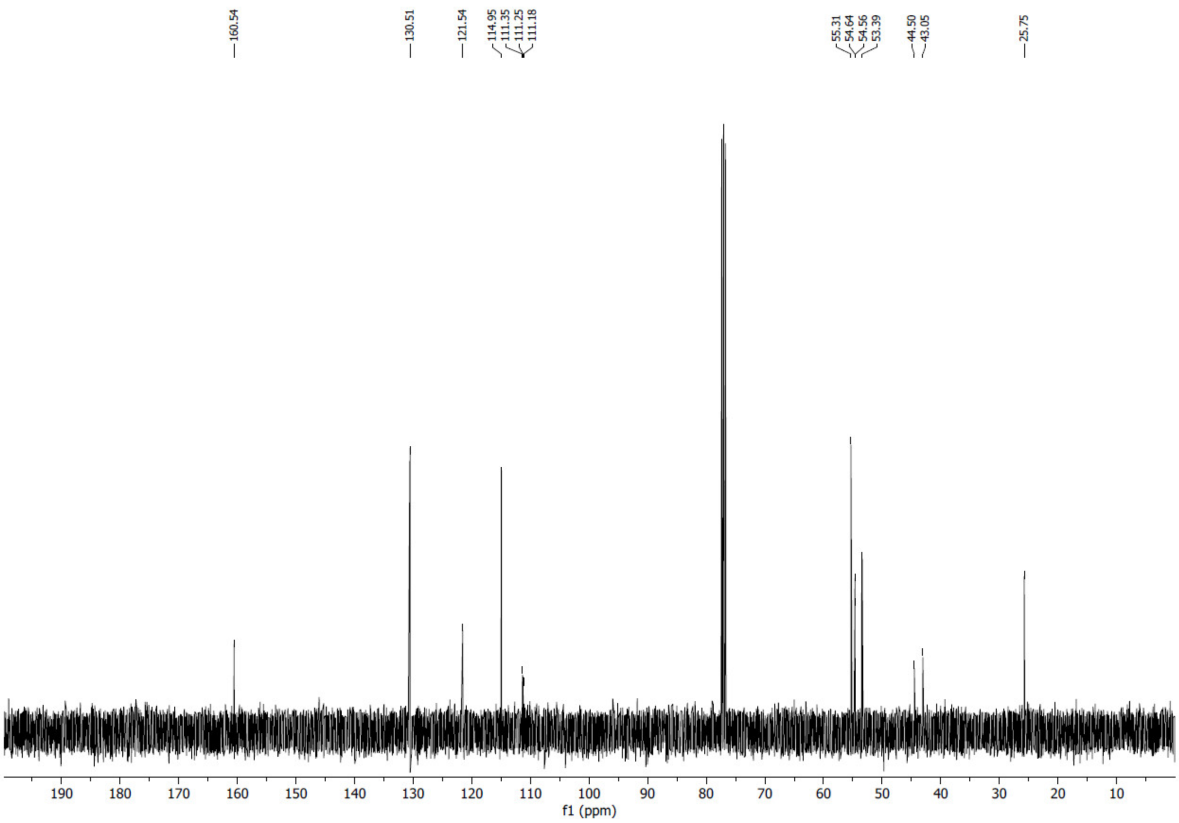

Figure S8. ^{13}CNMR (100 MHz, $CDCl_3$) spectra of dimethyl (2,2-dicyano-1-(4-methoxyphenyl)ethyl)phosphonate (**3**)

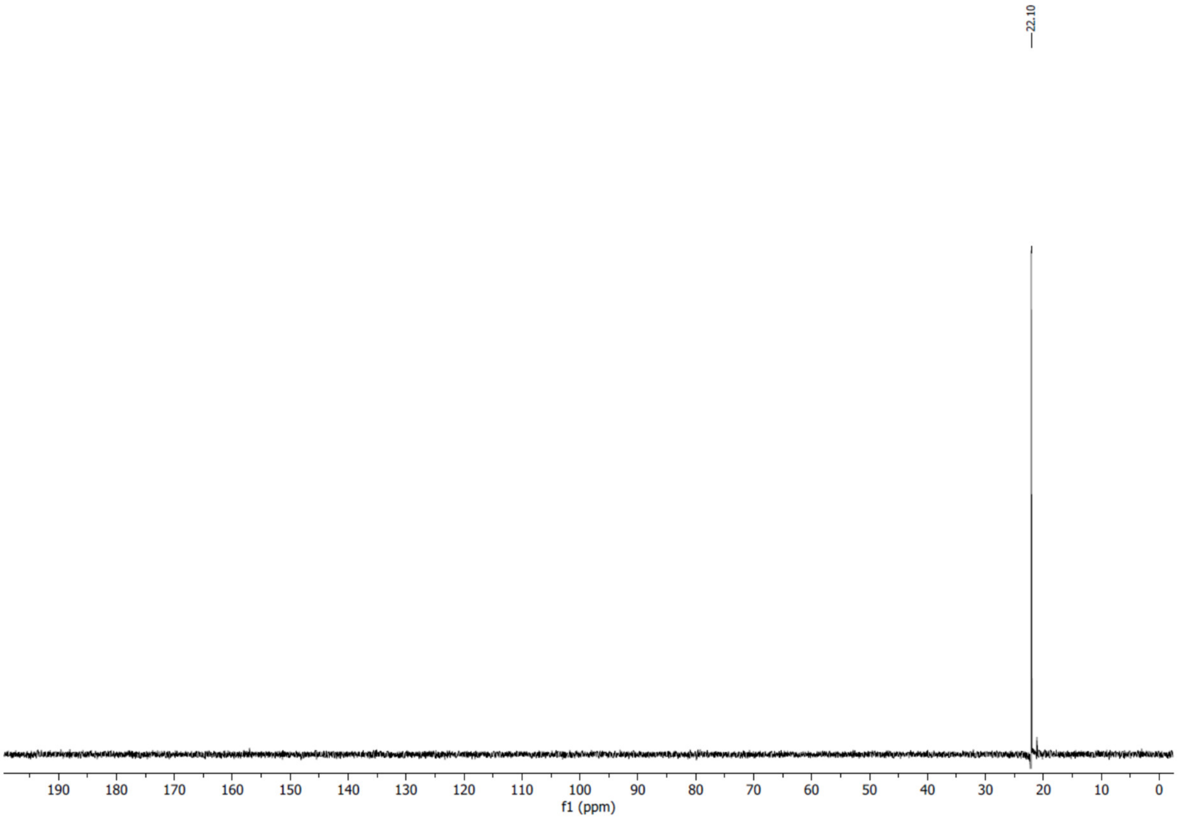

Figure S9. ^{31}PNMR (162 MHz, $CDCl_3$) spectra of dimethyl (2,2-dicyano-1-(4-methoxyphenyl)ethyl)phosphonate (**3**)

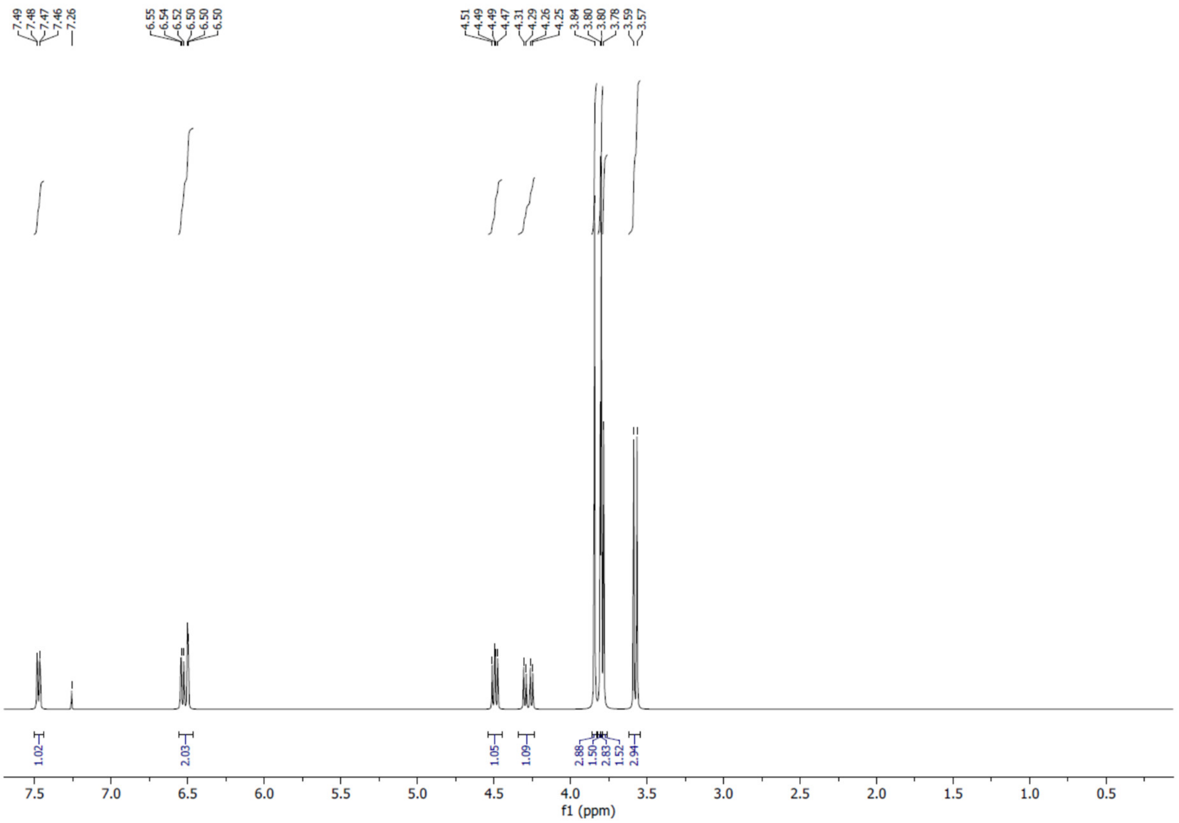

Figure S10. ^{1}HNMR (500 MHz, $CDCl_3$) spectra of dimethyl (2,2-dicyano-1-(2,4-dimethoxyphenyl)ethyl)phosphonate (**4**)

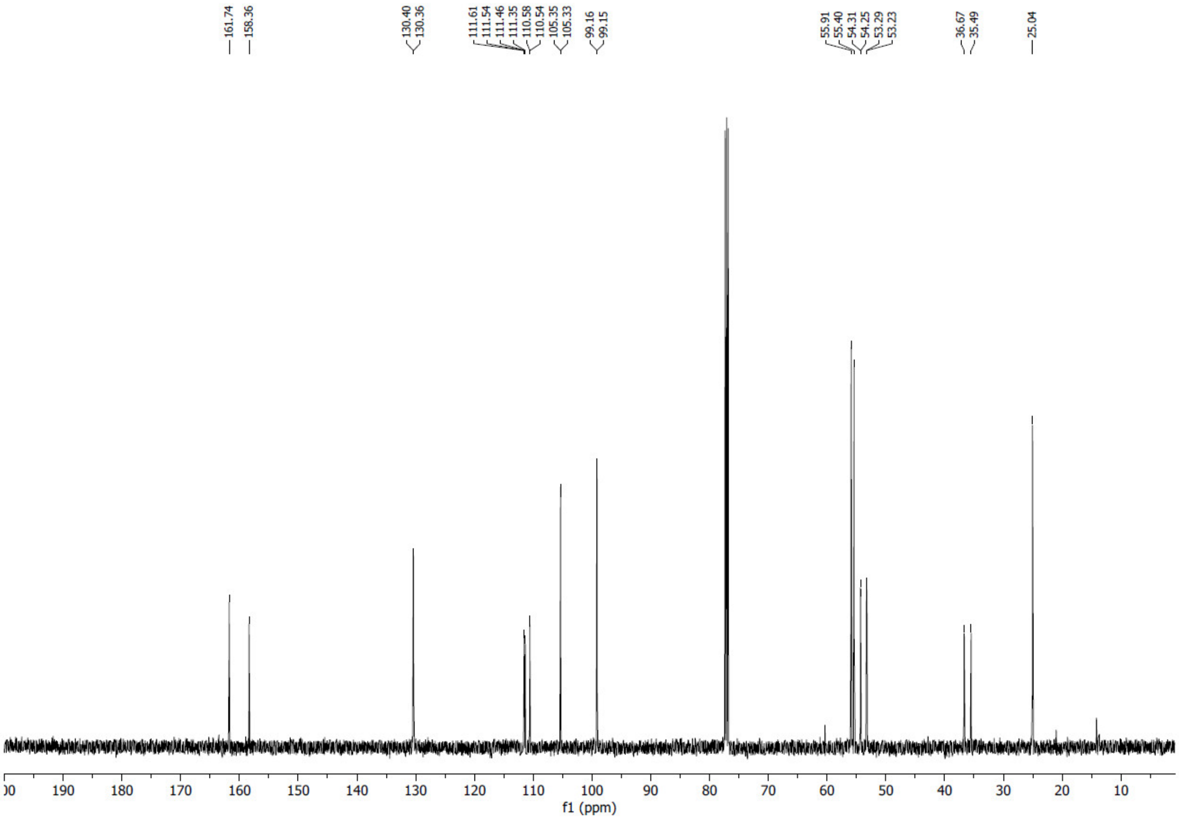

Figure S11. ^{13}CNMR (125 MHz, $CDCl_3$) spectra of dimethyl (2,2-dicyano-1-(2,4-dimethoxyphenyl)ethyl)phosphonate (**4**)

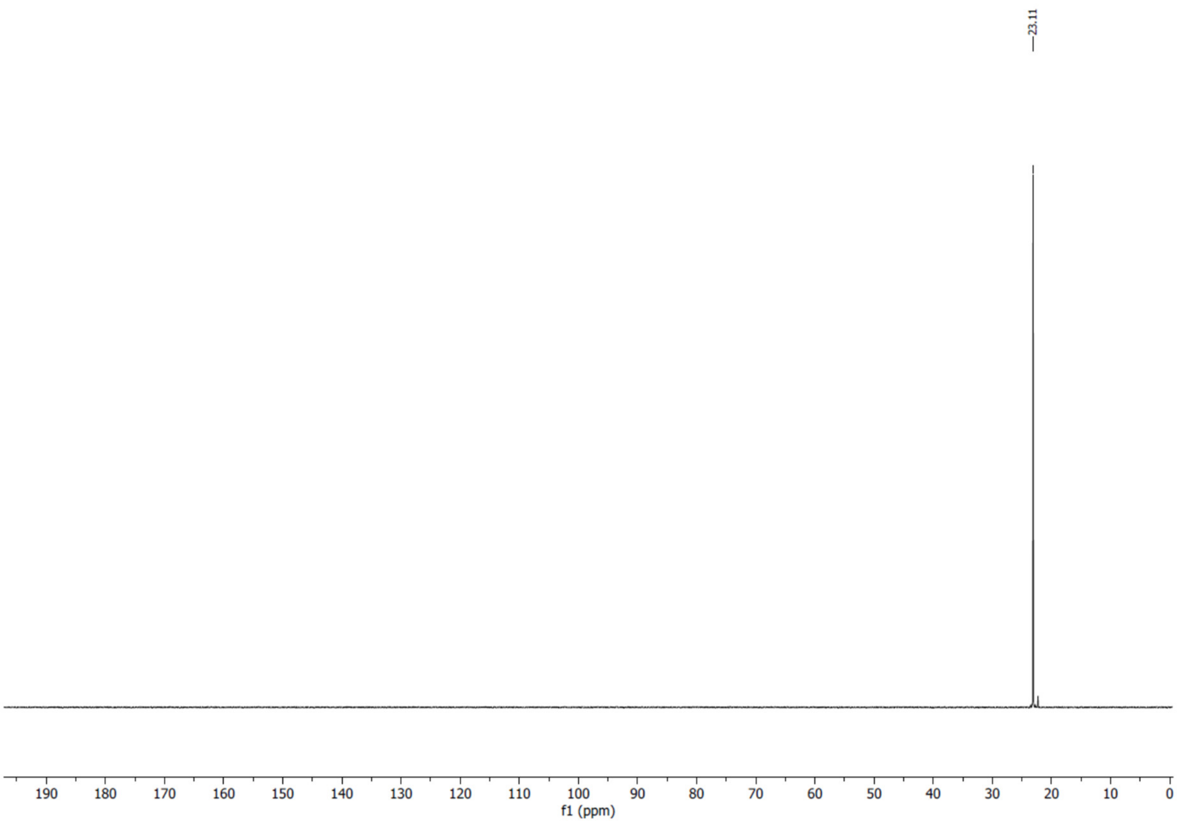

Figure S12. ^{31}PNMR (203 MHz, $CDCl_3$) spectra of dimethyl (2,2-dicyano-1-(2,4-dimethoxyphenyl)ethyl)phosphonate (**4**)

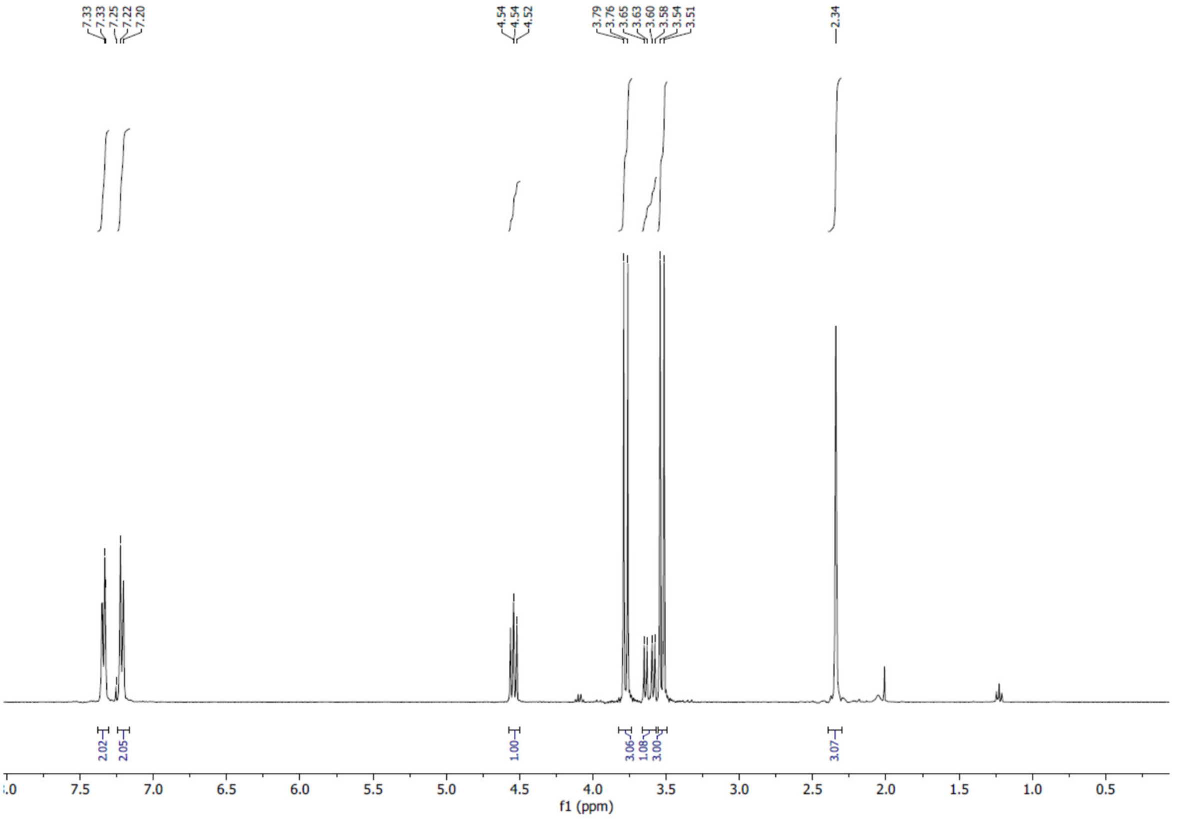

Figure S13. ^{1}H NMR (400 MHz, $CDCl_3$) spectra of dimethyl (2,2-dicyano-1-(4-methylphenyl)ethyl)phosphonate (**5**).

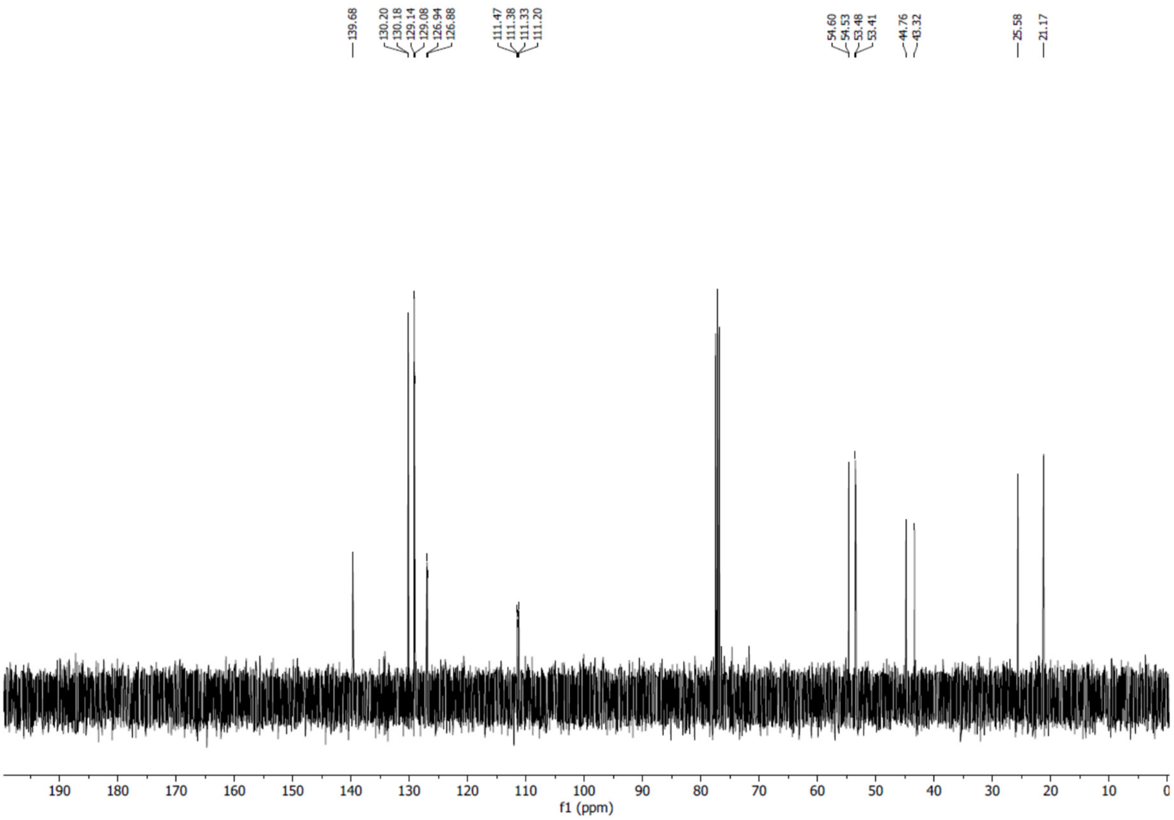

Figure S14. ^{13}C NMR (100 MHz, $CDCl_3$) spectra of dimethyl (2,2-dicyano-1-(4-methylphenyl)ethyl)phosphonate (**5**).

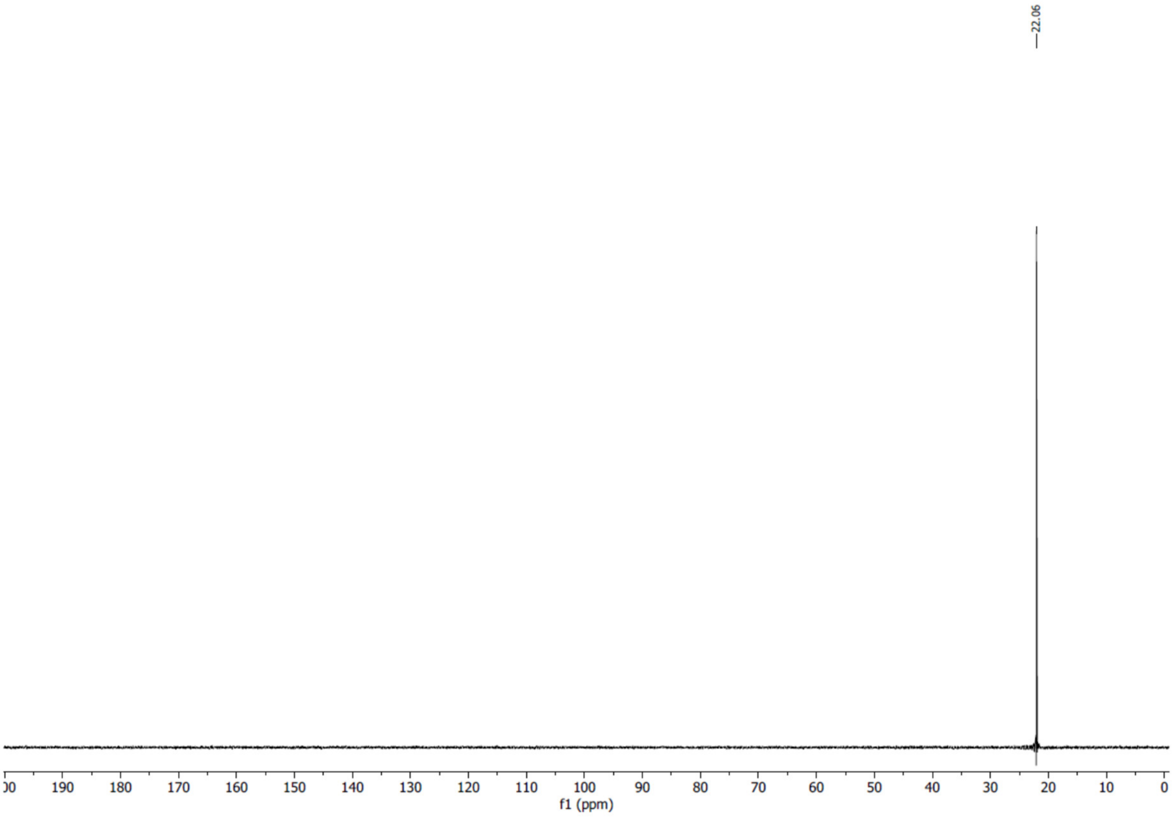

Figure S15. ^{31}P NMR (162 MHz, $CDCl_3$) spectra of dimethyl (2,2-dicyano-1-(4-methylphenyl)ethyl)phosphonate (**5**).

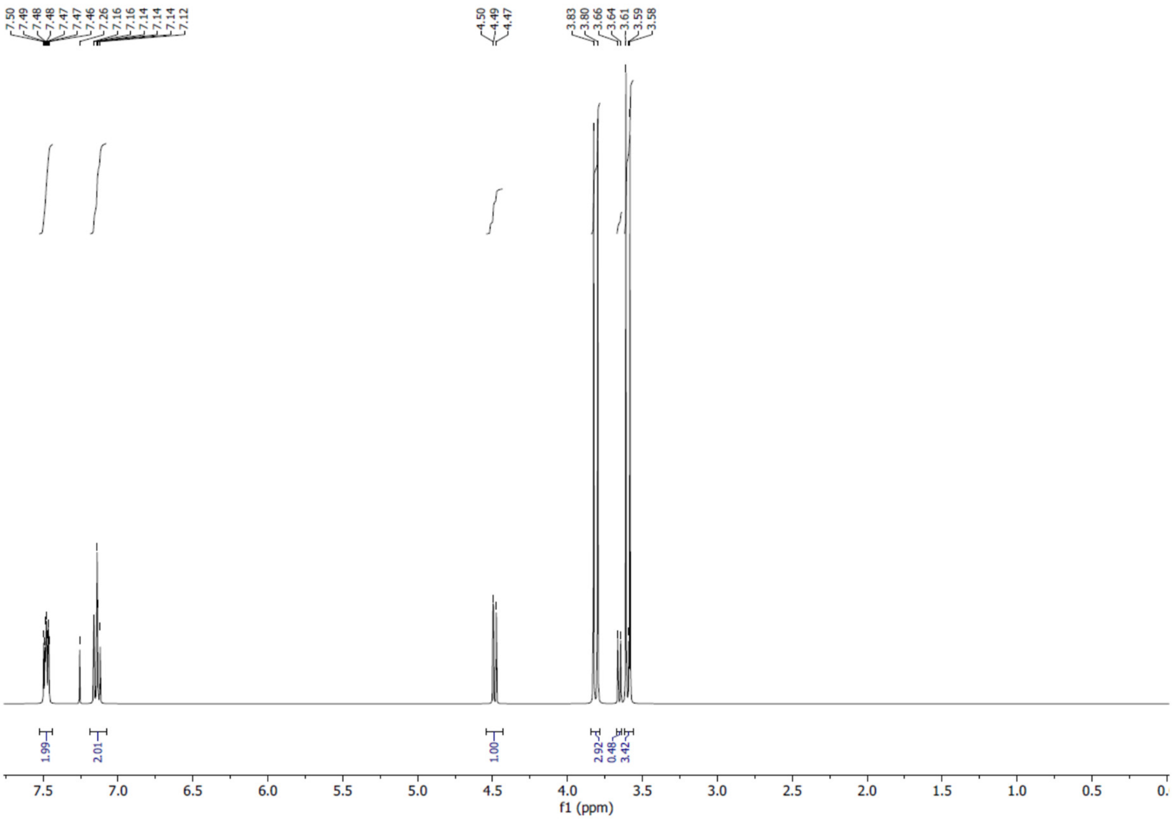

Figure S16. ¹H NMR (400 MHz, $CDCl_3$) spectra of dimethyl (2,2-dicyano-1-(4-fluorophenyl)ethyl)phosphonate (**6**).

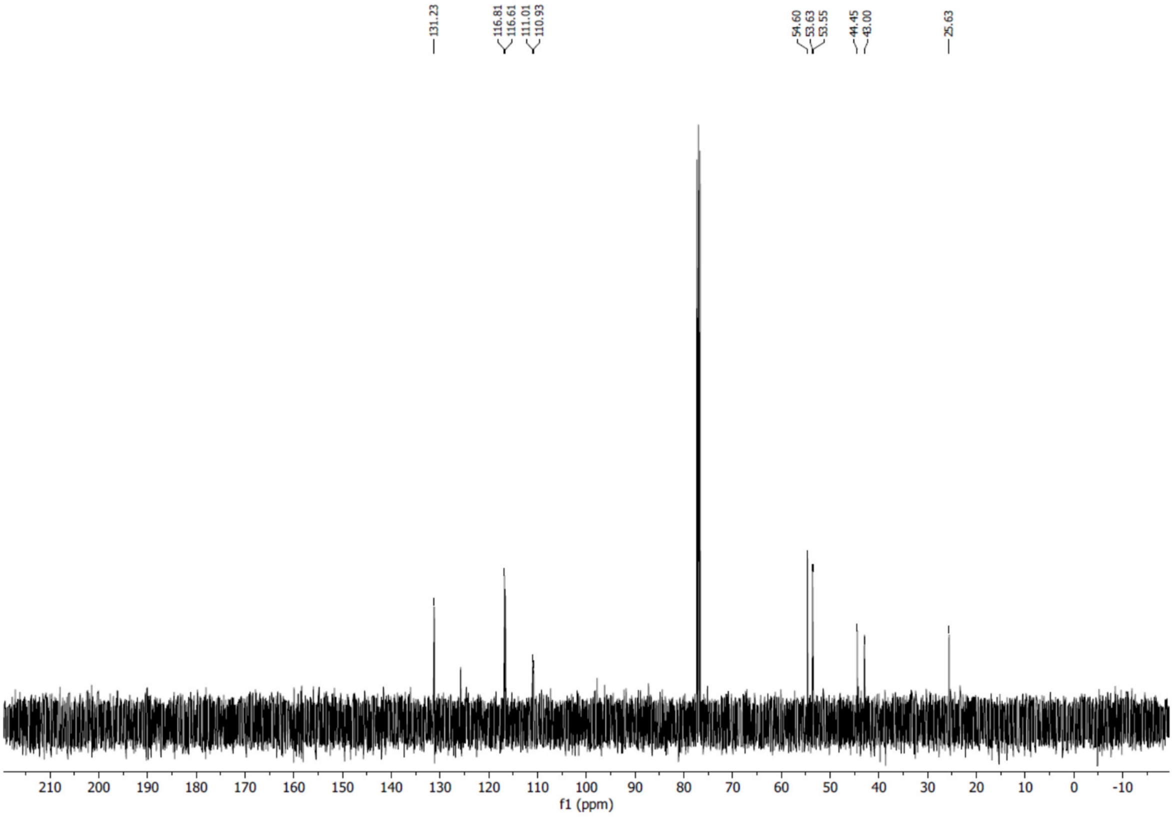

Figure S17. ¹³C NMR (100 MHz, $CDCl_3$) spectra of dimethyl (2,2-dicyano-1-(4-fluorophenyl)ethyl)phosphonate (**6**).

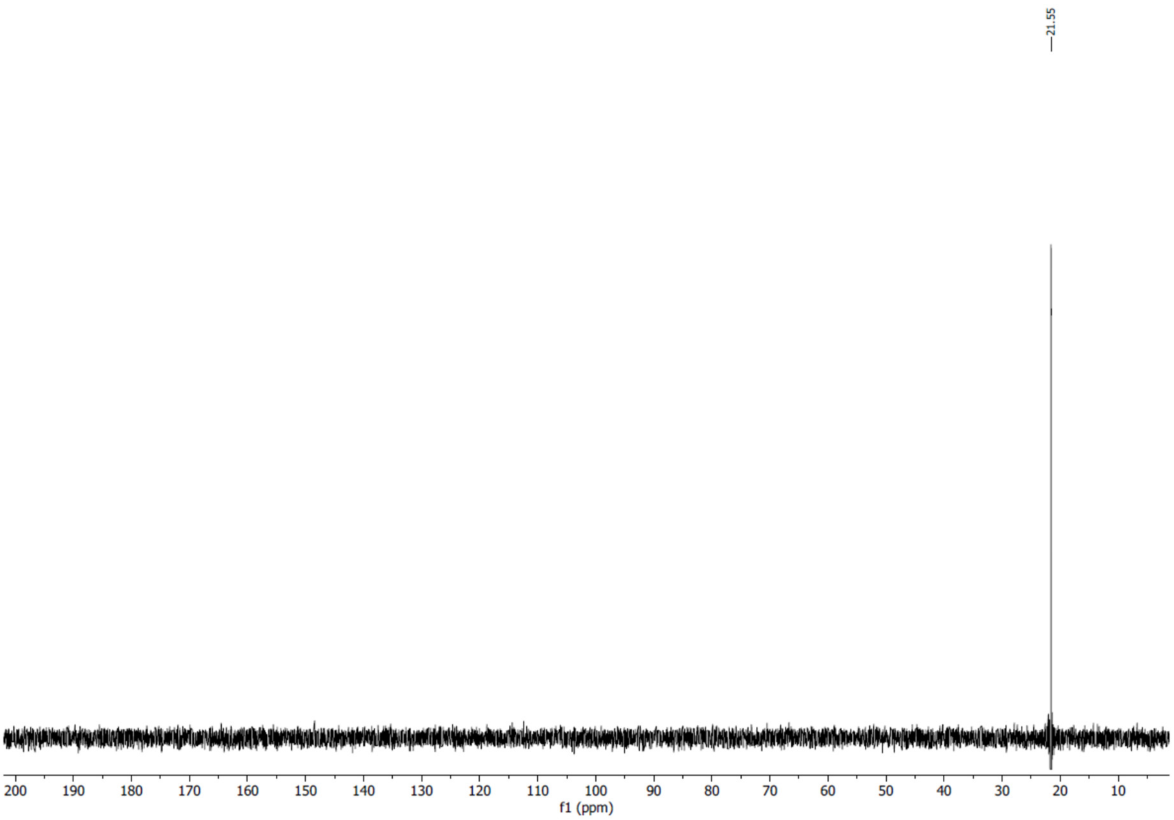

Figure S18. ^{31}P NMR (162 MHz, $CDCl_3$) spectra of dimethyl (2,2-dicyano-1-(4-fluorophenyl)ethyl)phosphonate (**6**).

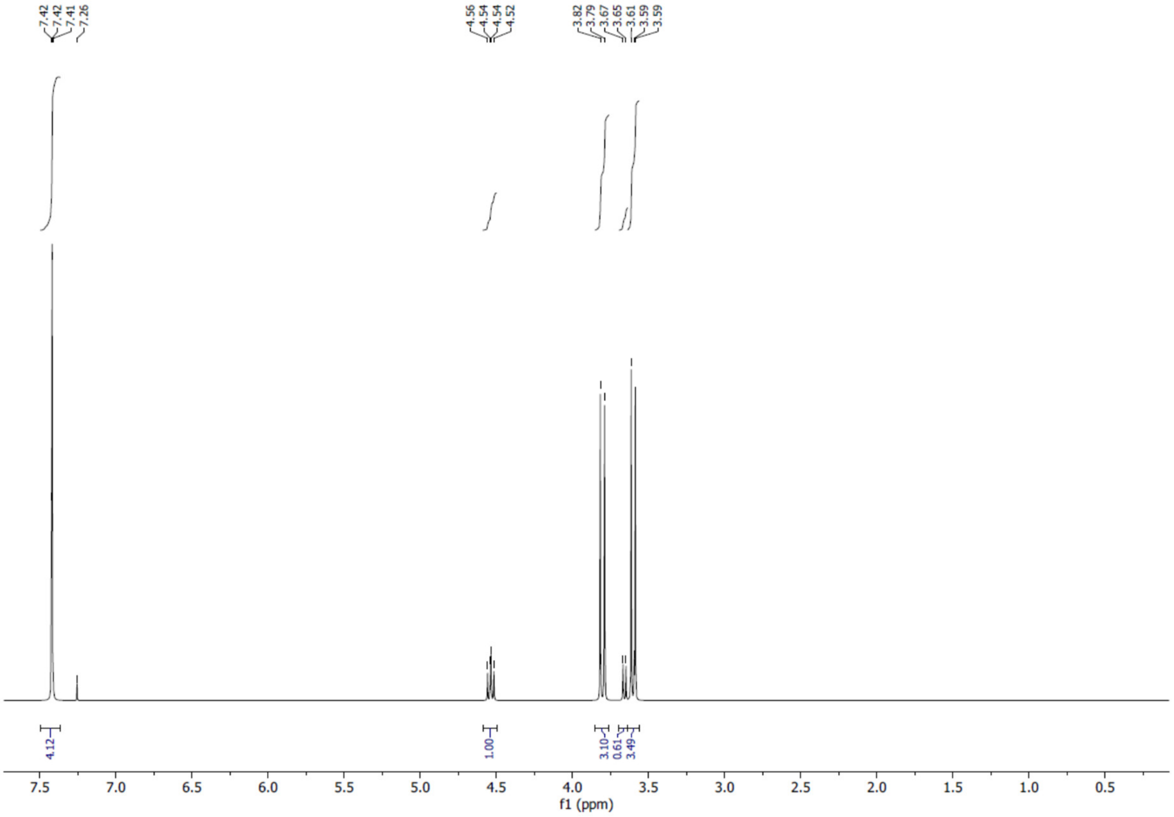

Figure S19. ^{1}H NMR (400 MHz, $CDCl_3$) spectra of dimethyl (2,2-dicyano-1-(4-chlorophenyl)ethyl)phosphonate (**7**).

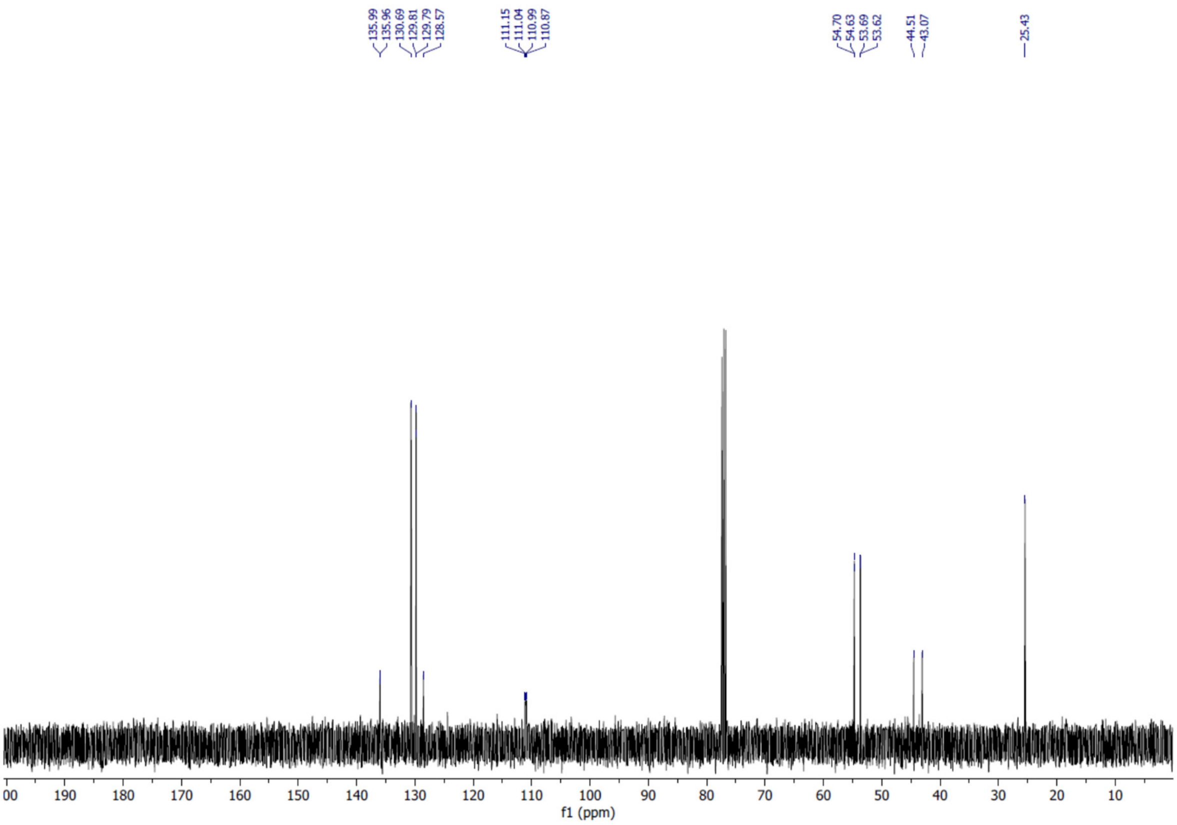

Figure S20. ^{13}C NMR (100 MHz, $CDCl_3$) spectra of dimethyl (2,2-dicyano-1-(4-chlorophenyl)ethyl)phosphonate (**7**).

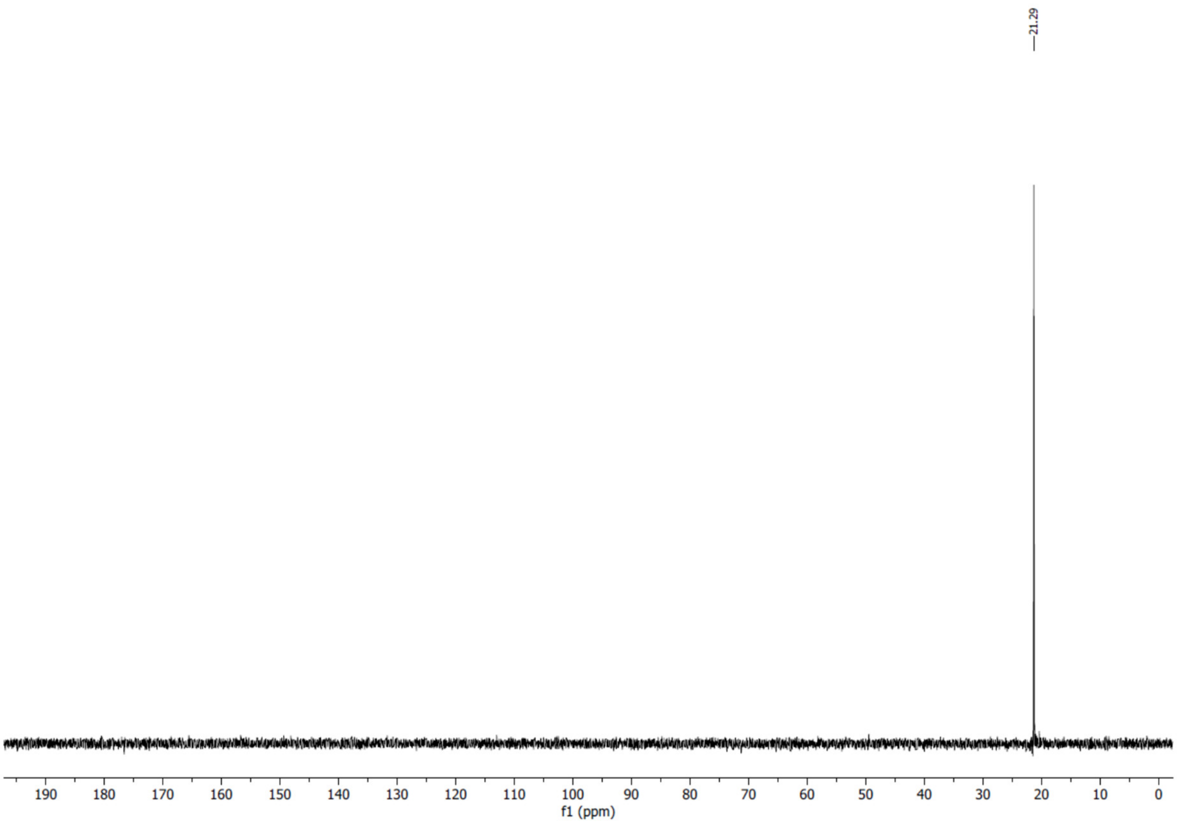

Figure S21. ^{31}P NMR (162 MHz, $CDCl_3$) spectra of dimethyl (2,2-dicyano-1-(4-chlorophenyl)ethyl)phosphonate (**7**).

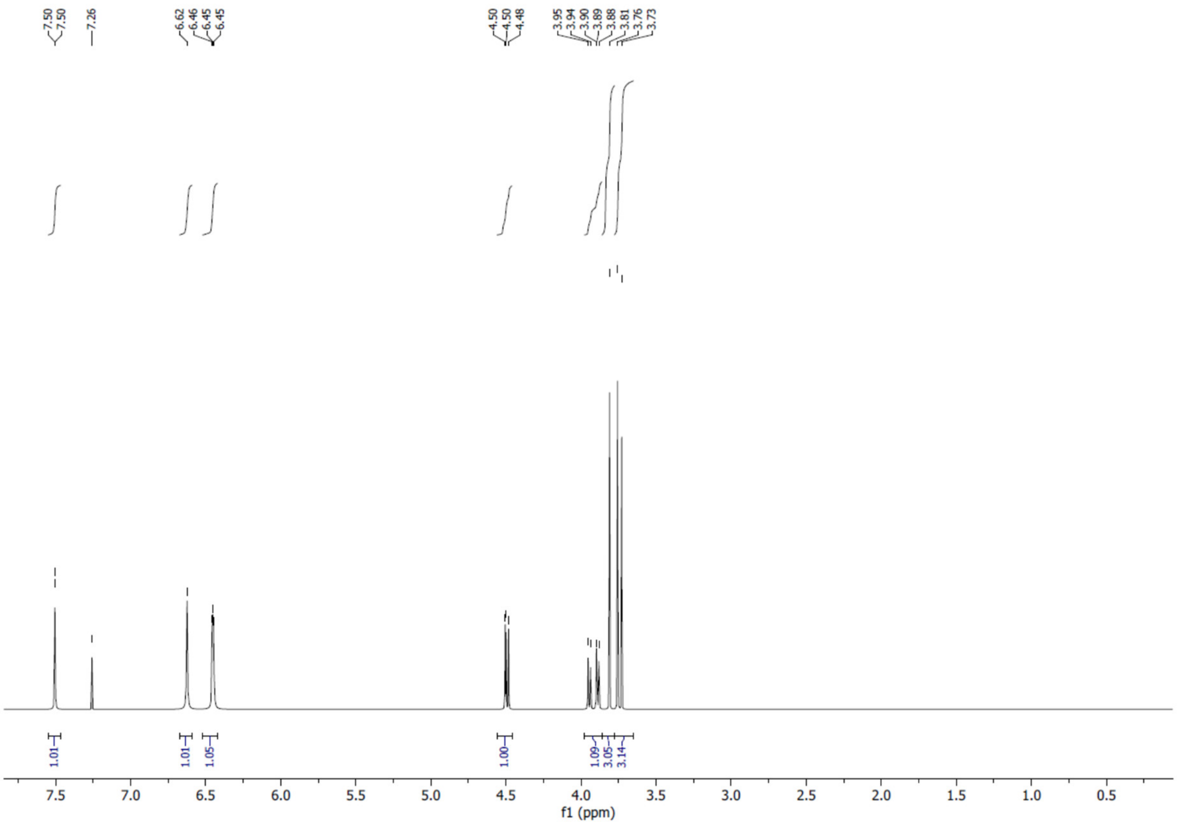

Figure S22. ^{1}H NMR (400 MHz, $CDCl_3$) spectra of dimethyl (2,2-dicyano-1-(furan-2-yl)ethyl)phosphonate (**8**).

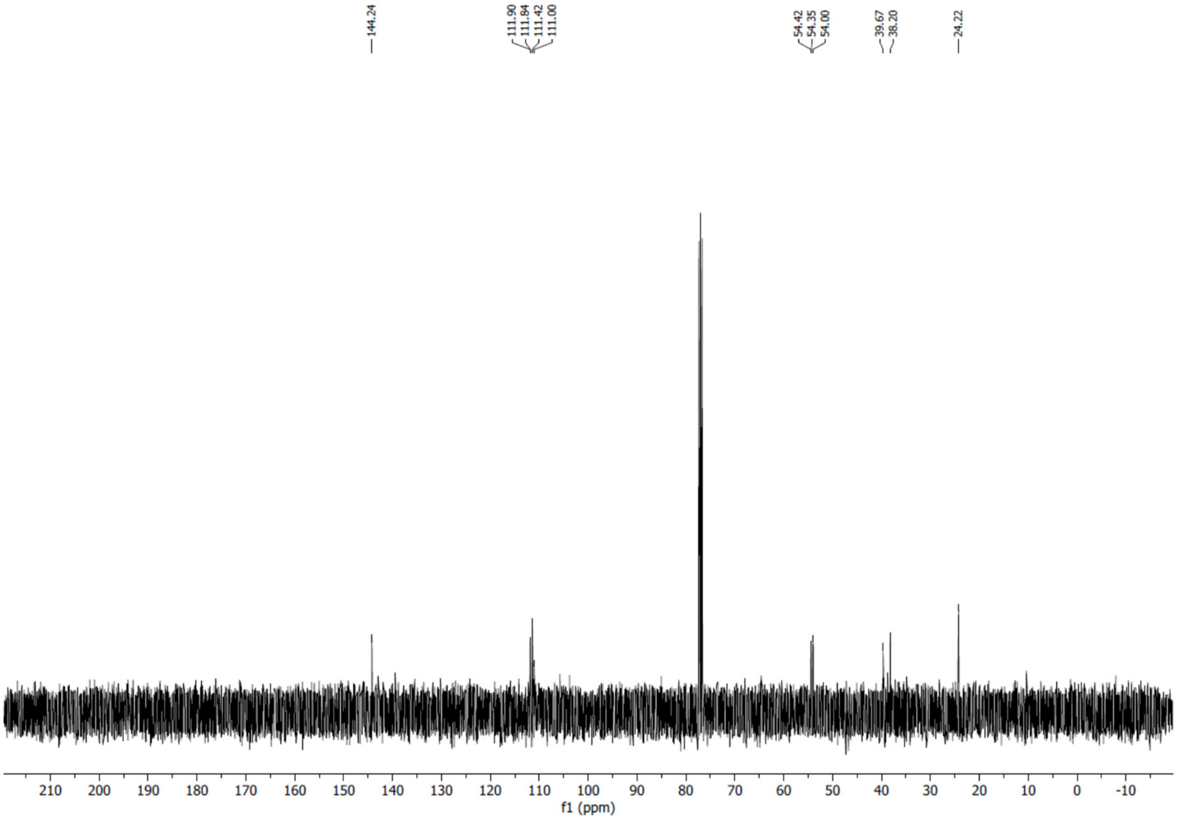

Figure S23. ^{13}C NMR (100 MHz, $CDCl_3$) spectra of dimethyl (2,2-dicyano-1-(furan-2-yl)ethyl)phosphonate (**8**).

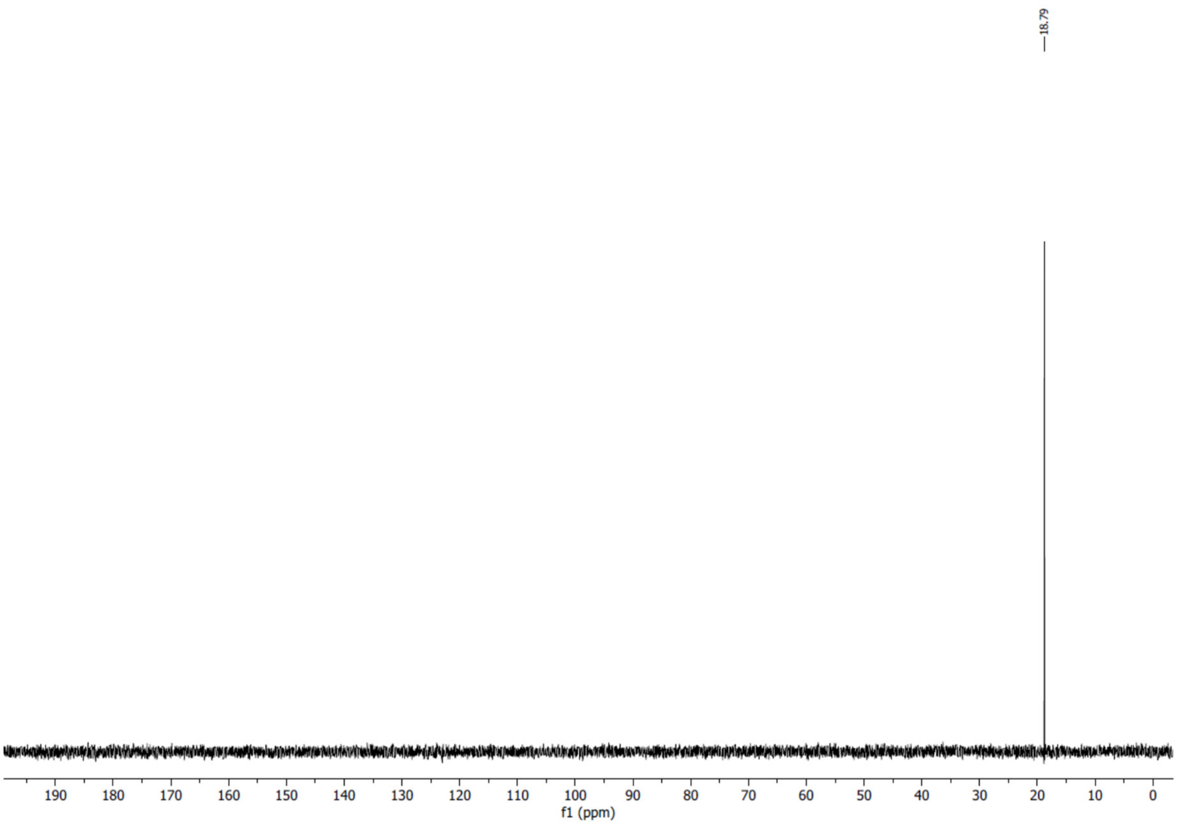

Figure S24. ^{31}P NMR (162 MHz, $CDCl_3$) spectra of dimethyl (2,2-dicyano-1-(furan-2-yl)ethyl)phosphonate (**8**).

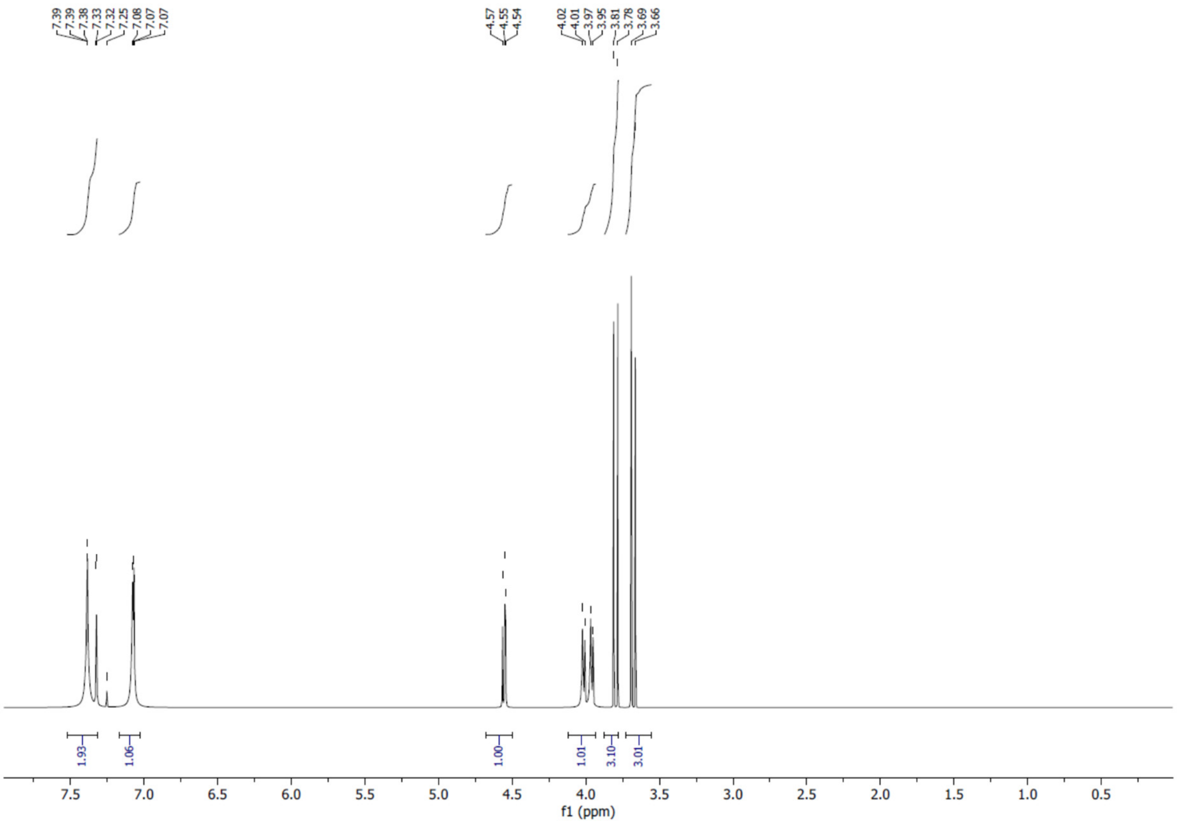

Figure S25. ^{1}H NMR (400 MHz, $CDCl_3$) spectra of dimethyl (2,2-dicyano-1-(thiophen-2-yl)ethyl)phosphonate (**9**).

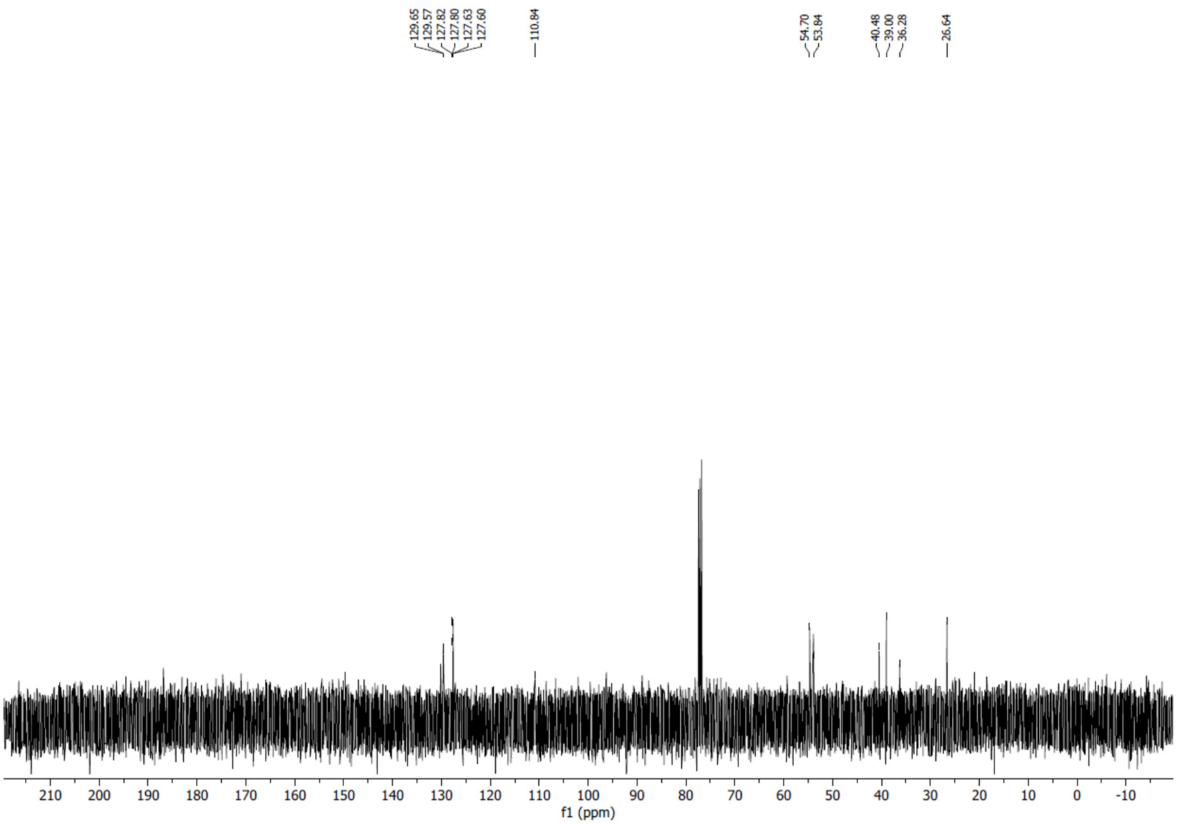

Figure S26. ^{13}C NMR (100 MHz, $CDCl_3$) spectra of dimethyl (2,2-dicyano-1-(thiophen-2-yl)ethyl)phosphonate (**9**).

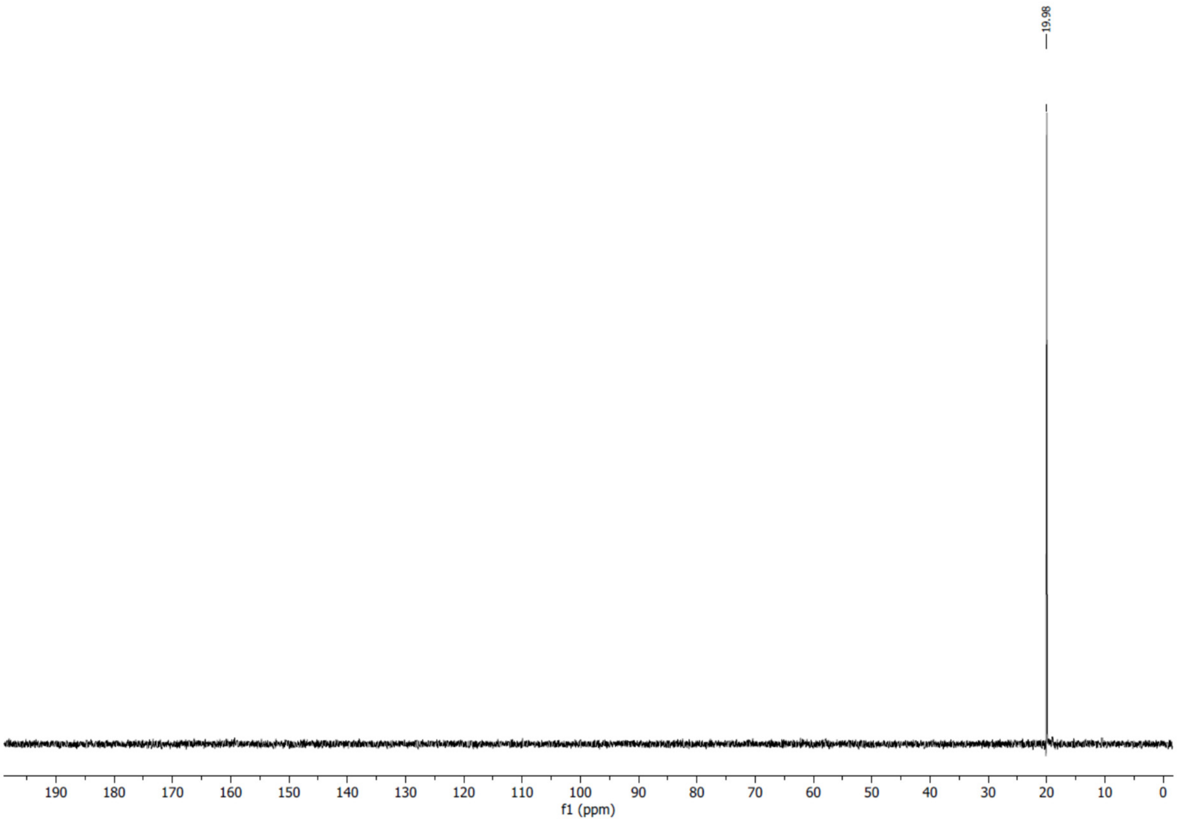

Figure S27. ^{31}P NMR (162 MHz, $CDCl_3$) spectra of dimethyl (2,2-dicyano-1-(thiophen-2-yl)ethyl)phosphonate (**9**).

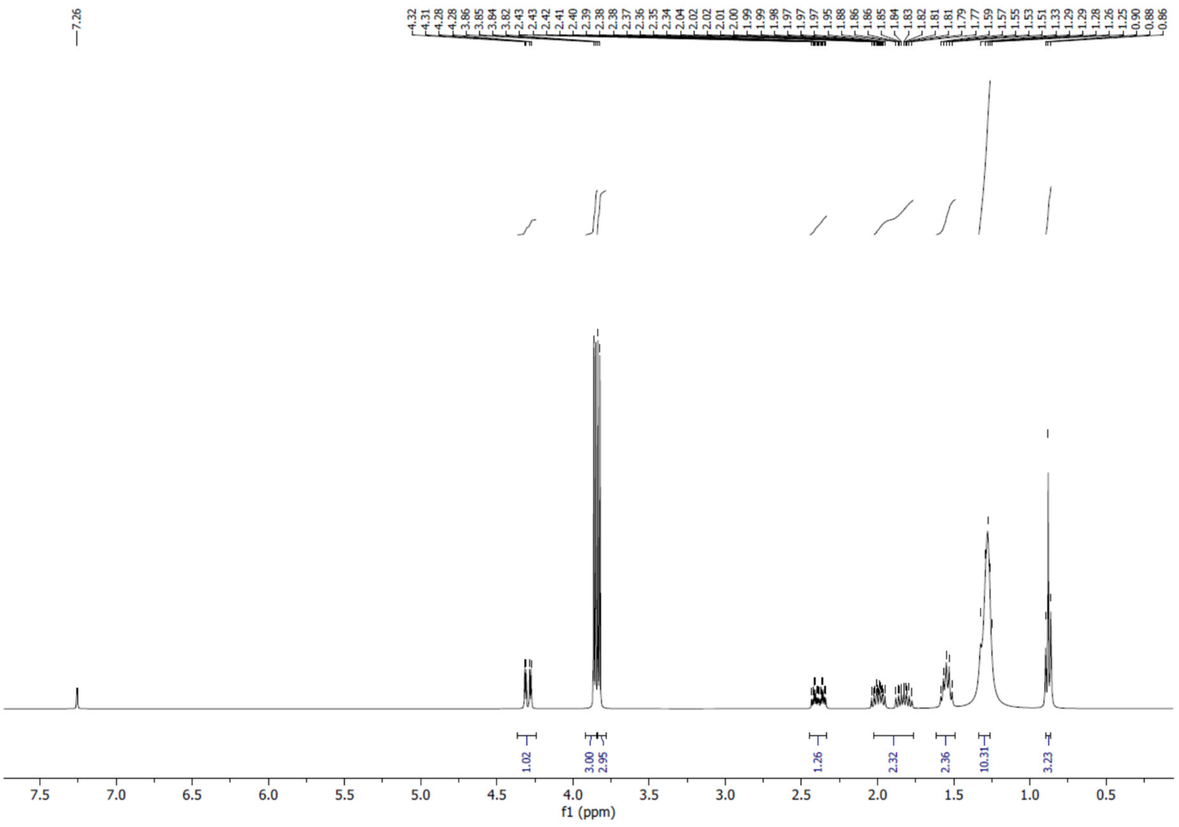

Figure S28. ^{1}H NMR (400 MHz, $CDCl_3$) spectra of dimethyl (2,2-dicyano-1-monooctyl)phosphonate (**10**).

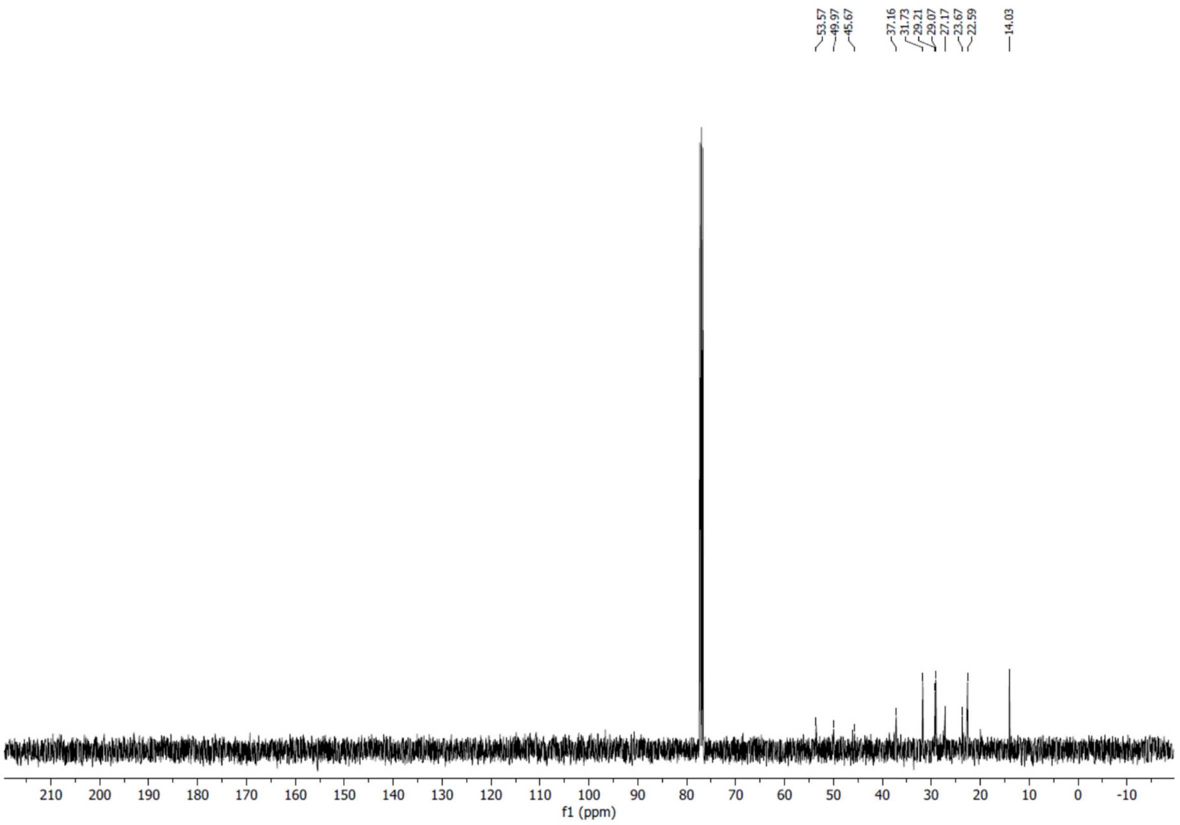

Figure S29. ^{13}C NMR (100 MHz, $CDCl_3$) spectra of dimethyl (2,2-dicyano-1-monooctyl)phosphonate (**10**).

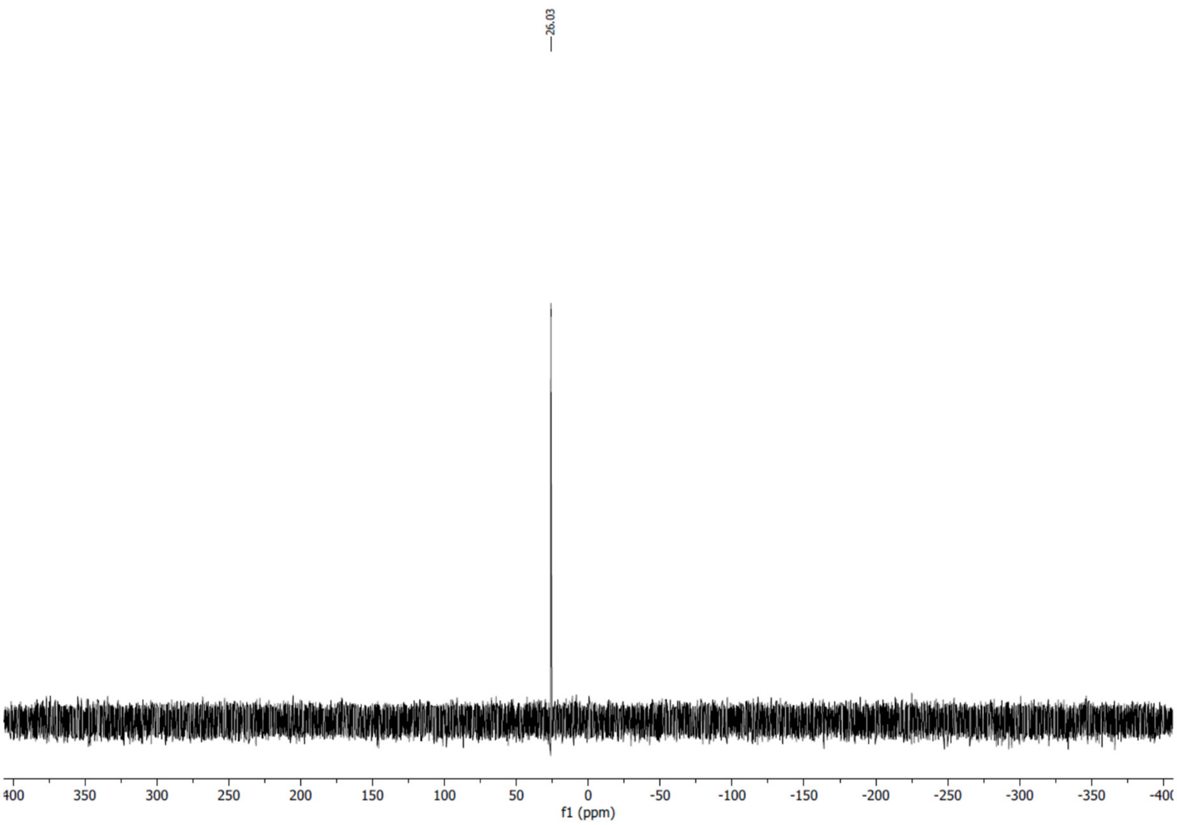

Figure S30. ^{31}P NMR (162 MHz, $CDCl_3$) spectra of dimethyl (2,2-dicyano-1-monooctyl)phosphonate (**10**).

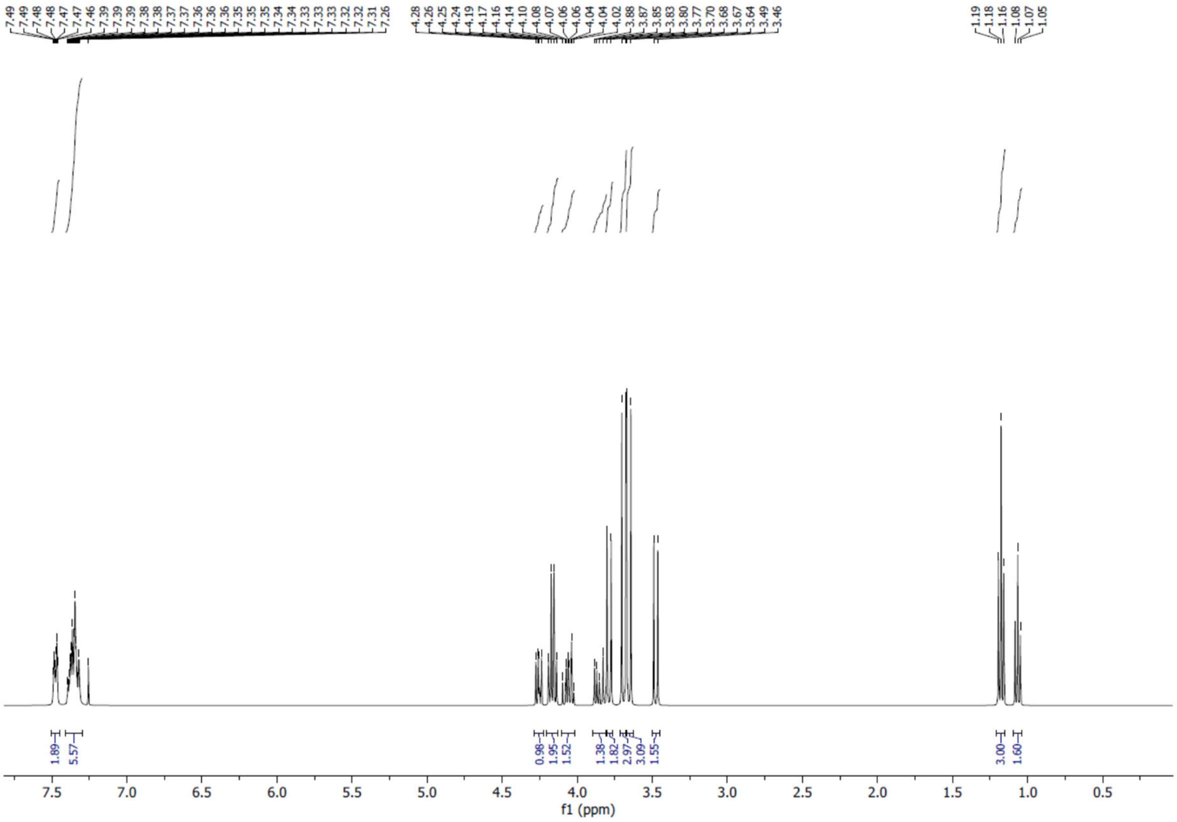

Figure S31. ^{1}H NMR (400 MHz, $CDCl_3$) spectra of 2-cyano-3-(dimethoxy-phosphoryl)-3-phenyl-propionic acid ethyl ester (**11**).

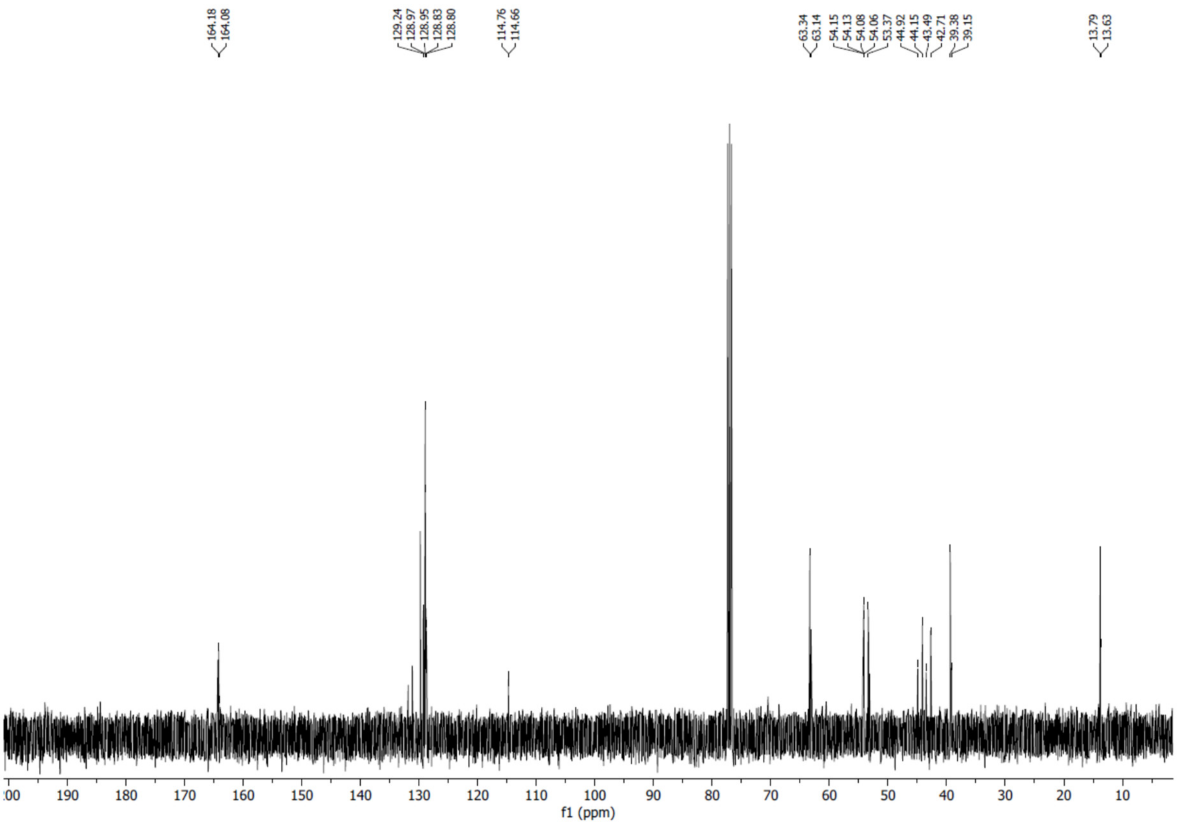

Figure S32. ^{13}C NMR (100 MHz, $CDCl_3$) spectra of 2-cyano-3-(dimethoxy-phosphoryl)-3-phenyl-propionic acid ethyl ester (**11**).

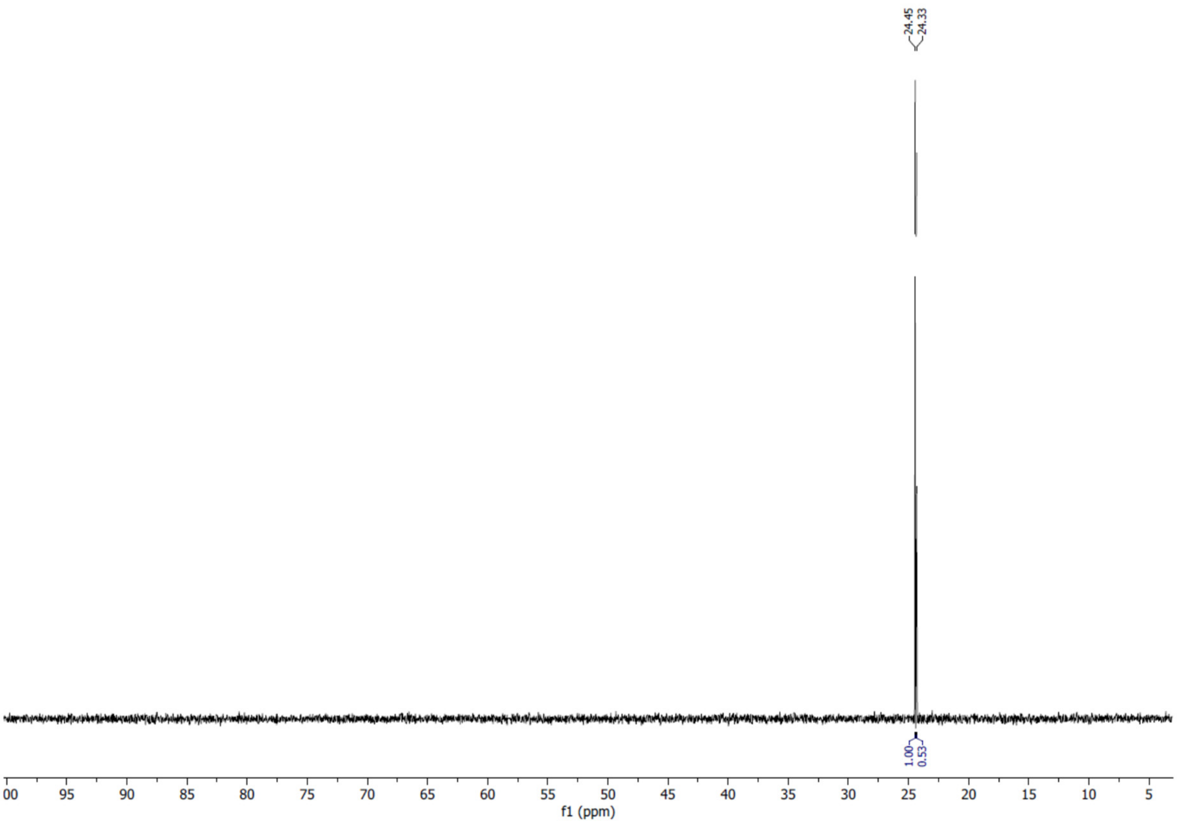

Figure S33. ^{31}P NMR (162 MHz, $CDCl_3$) spectra of 2-cyano-3-(dimethoxy-phosphoryl)-3-phenyl-propionic acid ethyl ester (**11**).

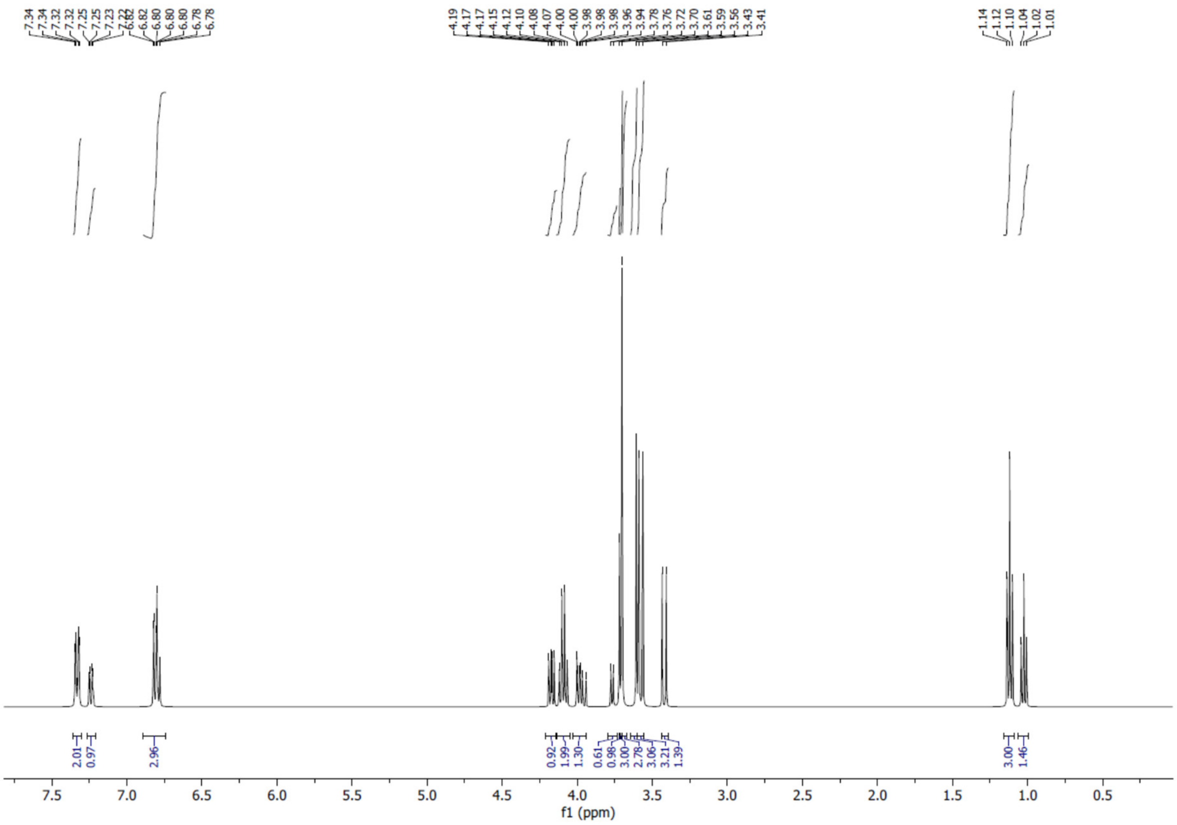

Figure S34. ^{1}H NMR (400 MHz, $CDCl_3$) spectra of 2-cyano-3-(dimethoxy-phosphoryl)-3-(4-methoxyphenyl)-propionic acid ethyl ester (**12**).

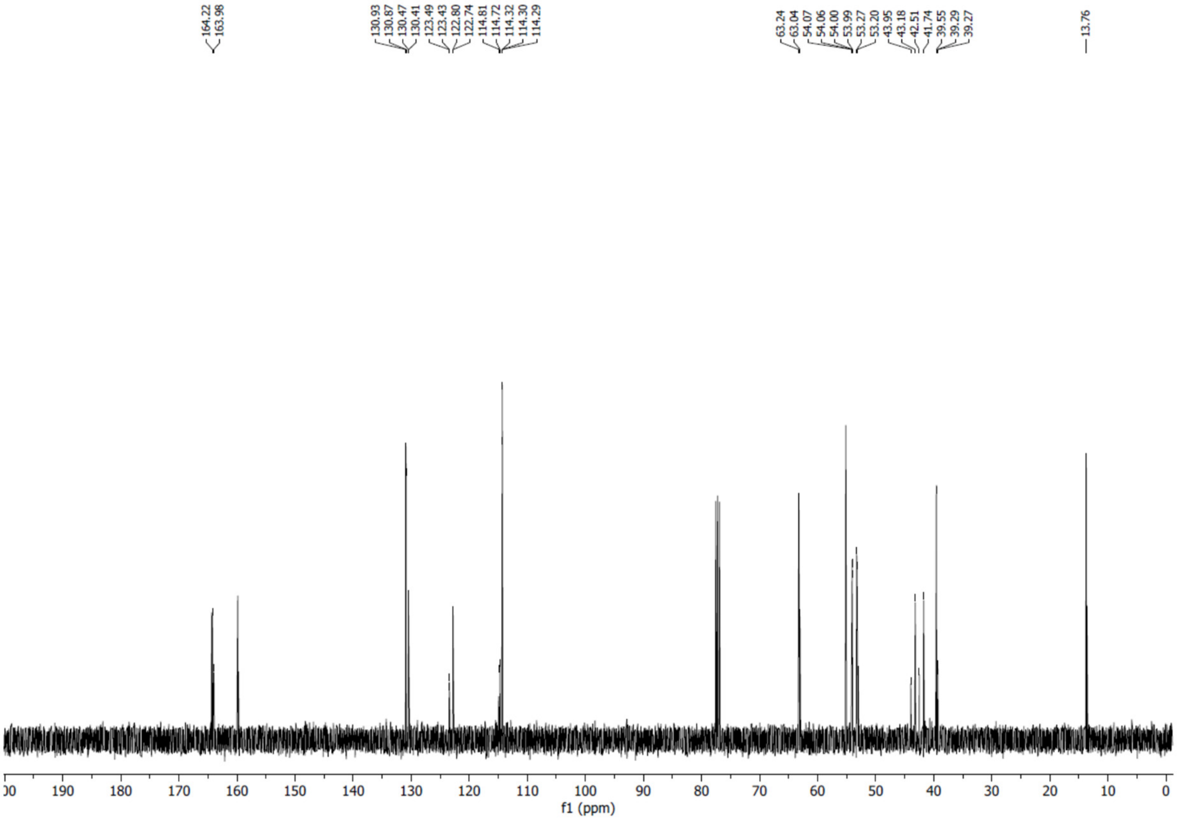

Figure S35. ^{13}C NMR (100 MHz, $CDCl_3$) spectra of 2-cyano-3-(dimethoxy-phosphoryl)-3-(4-methoxyphenyl)-propionic acid ethyl ester (**12**).

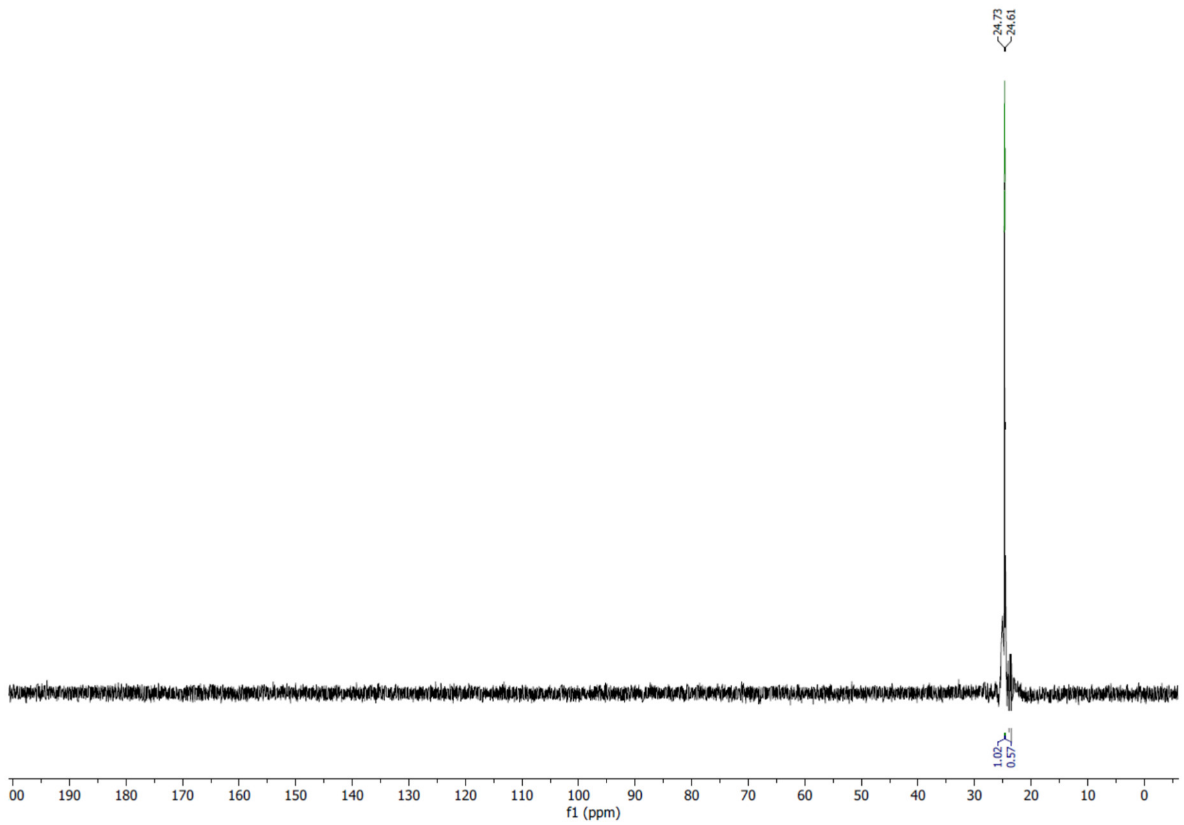

Figure S36. ^{31}P NMR (162 MHz, $CDCl_3$) spectra of 2-cyano-3-(dimethoxy-phosphoryl)-3-(4-methoxyphenyl)-propionic acid ethyl ester (**12**)

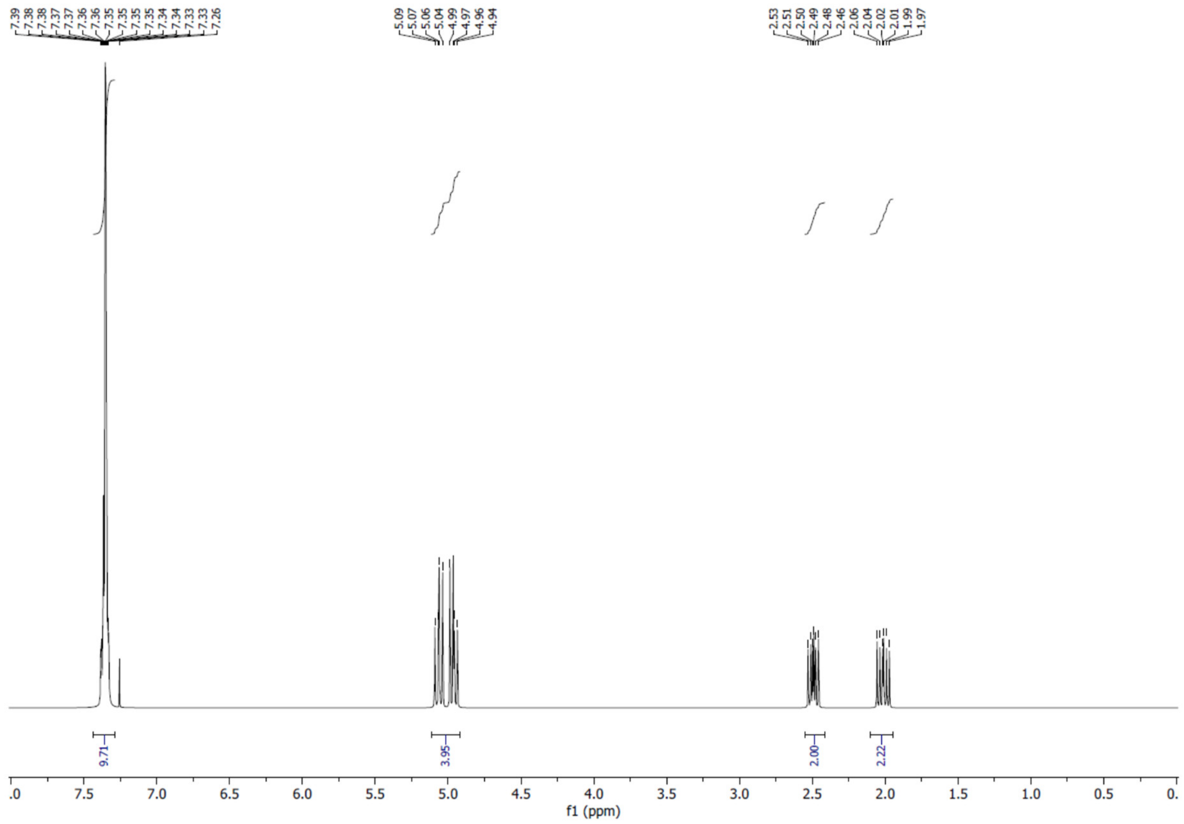

Figure S37. ^{1}H NMR (400 MHz, $CDCl_3$) spectra of dibenzyl (2-cyanoethyl)phosphonate (**13**).

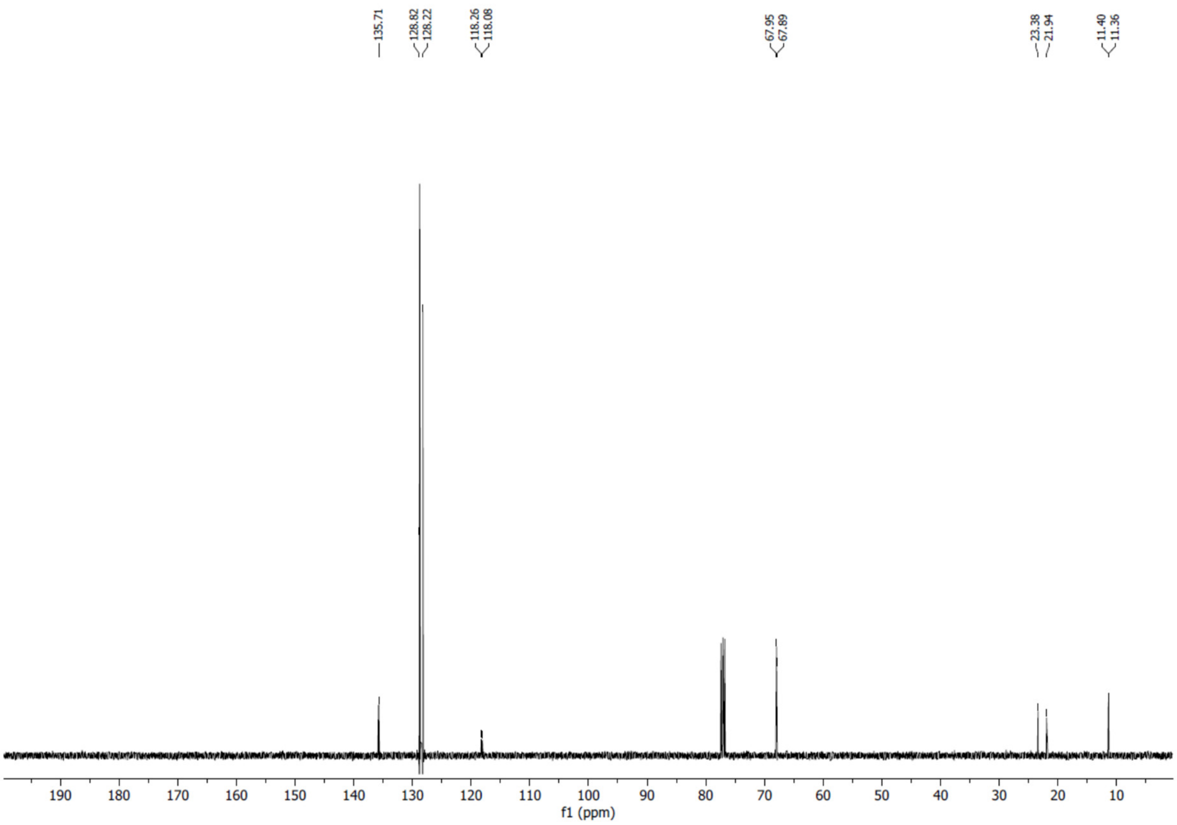

Figure S38. ^{13}C NMR (100 MHz, $CDCl_3$) spectra of dibenzyl (2-cyanoethyl)phosphonate (**13**).

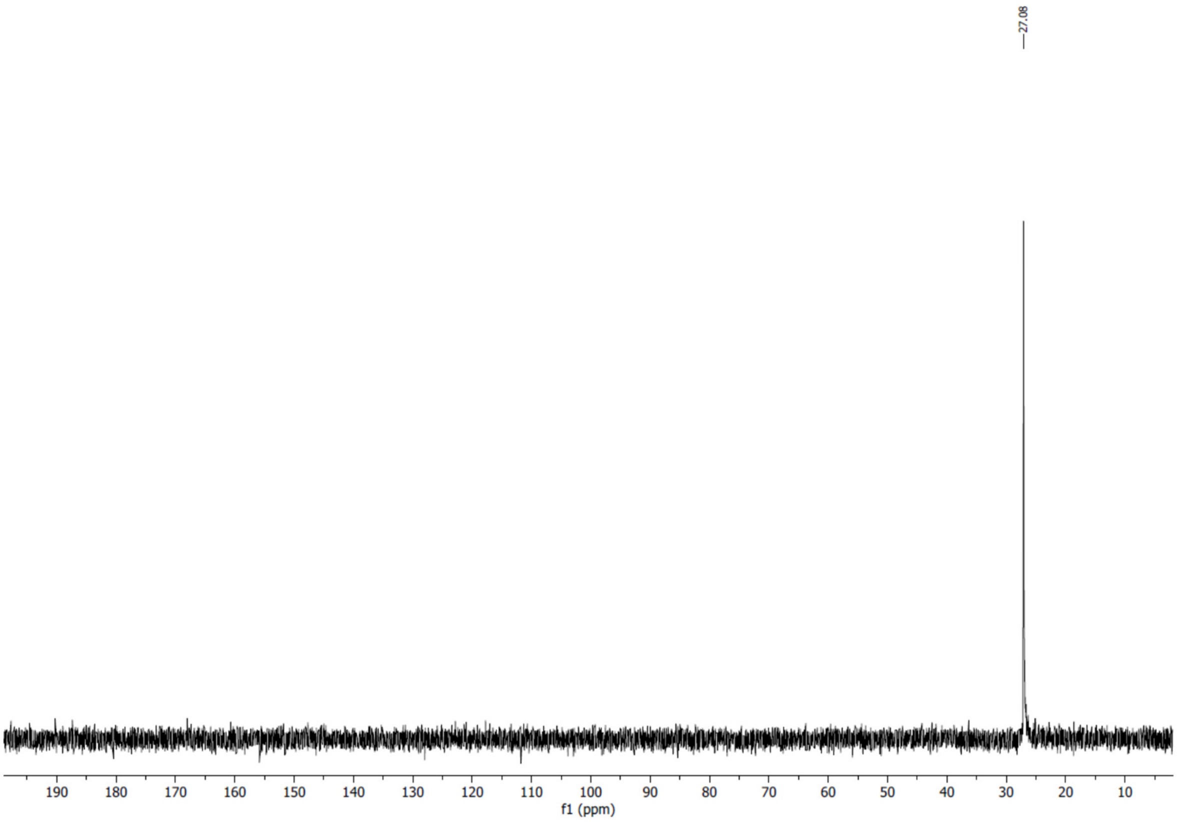

Figure S39. ^{31}P NMR (162 MHz, $CDCl_3$) spectra of dibenzyl (2-cyanoethyl)phosphonate (**13**).

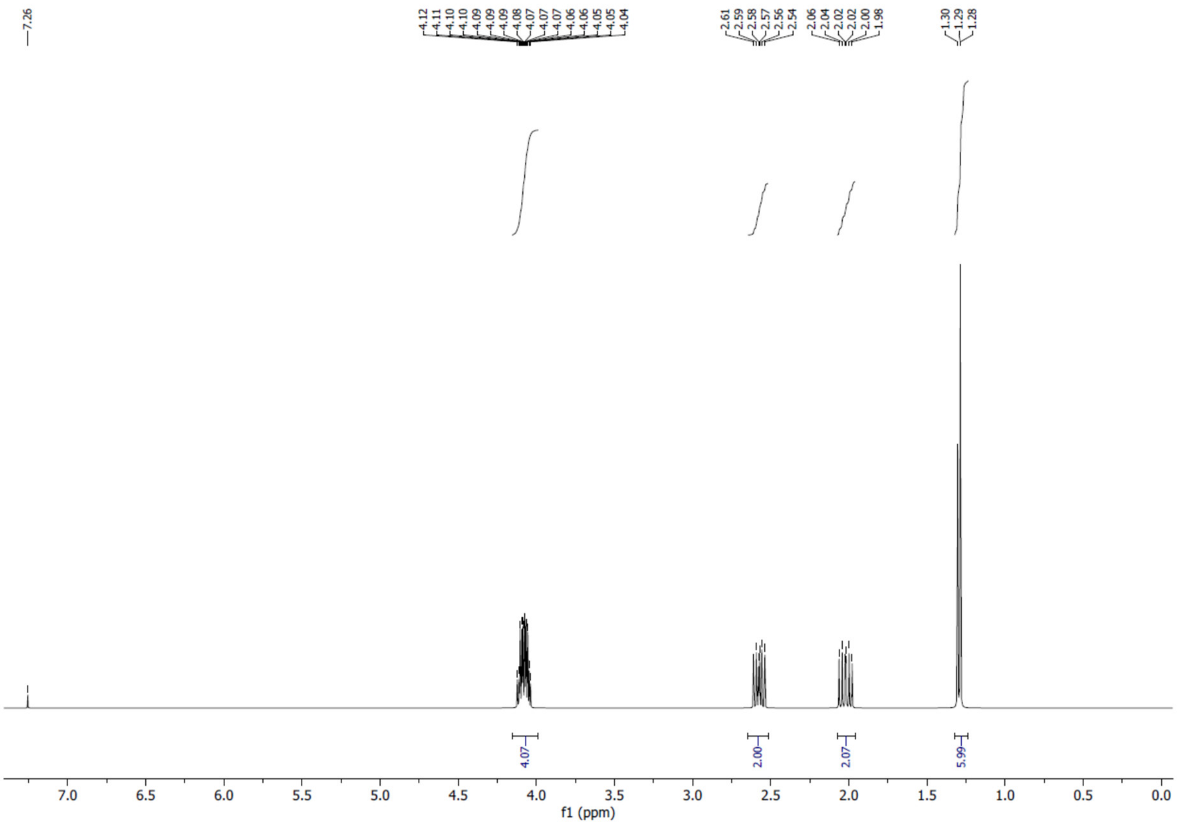

Figure S40. ^{1}H NMR (400 MHz, $CDCl_3$) spectra of diethyl (2-cyanoethyl)phosphonate (**14**).

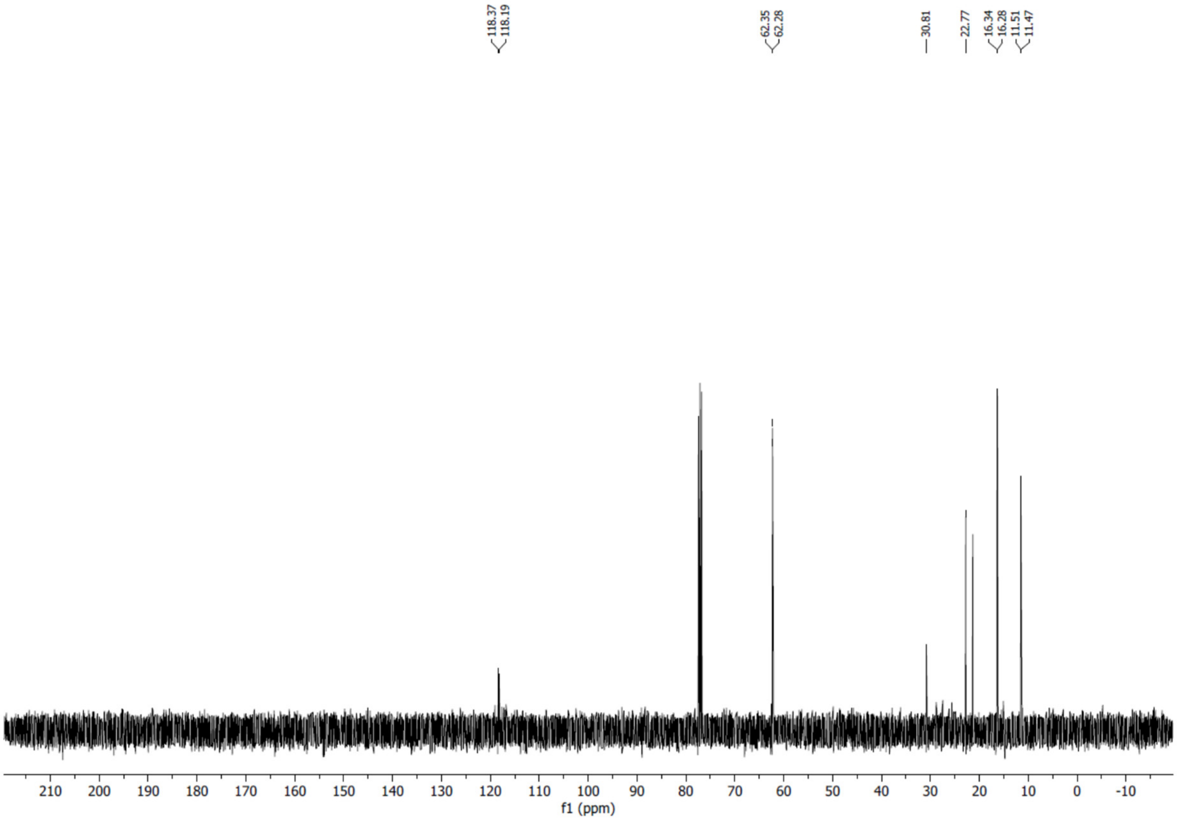

Figure S41. ^{13}C NMR (400 MHz, $CDCl_3$) spectra of diethyl (2-cyanoethyl)phosphonate (**14**).

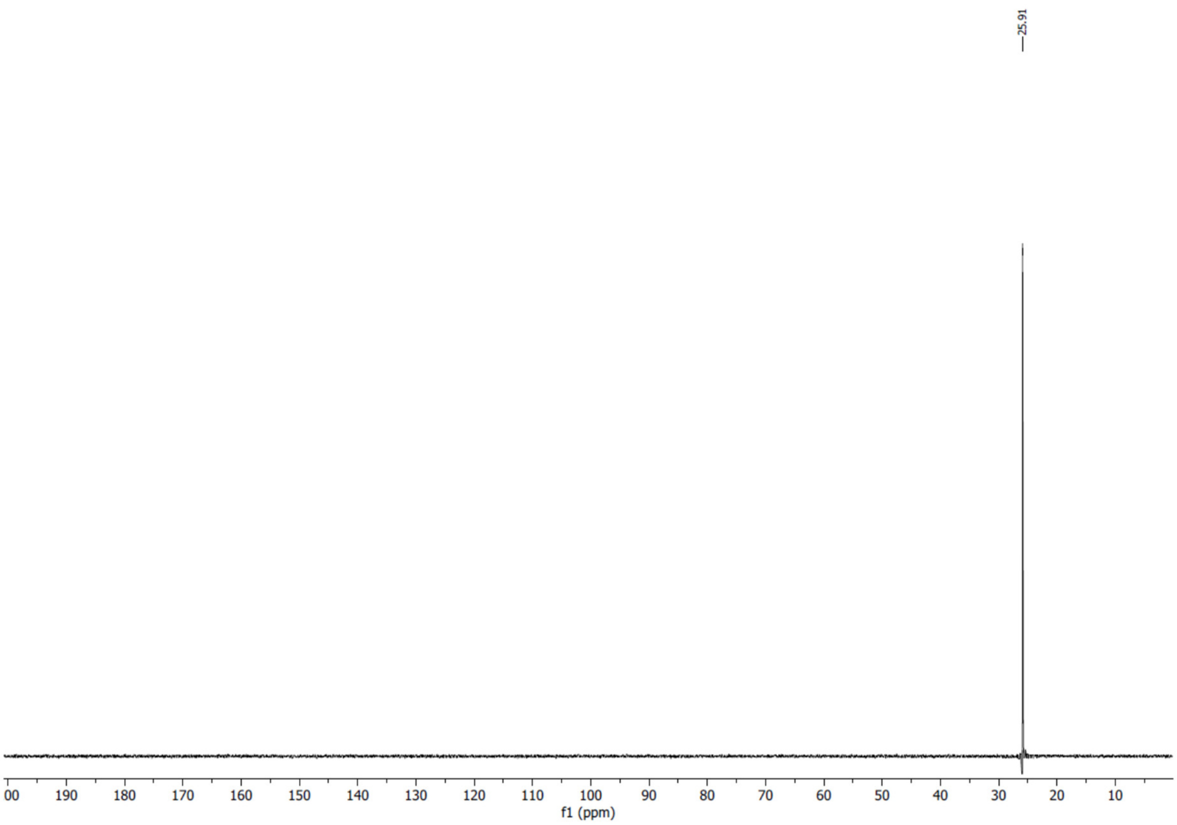

Figure S42. ^{31}P NMR (162 MHz, $CDCl_3$) spectra of diethyl (2-cyanoethyl)phosphonate (**14**).

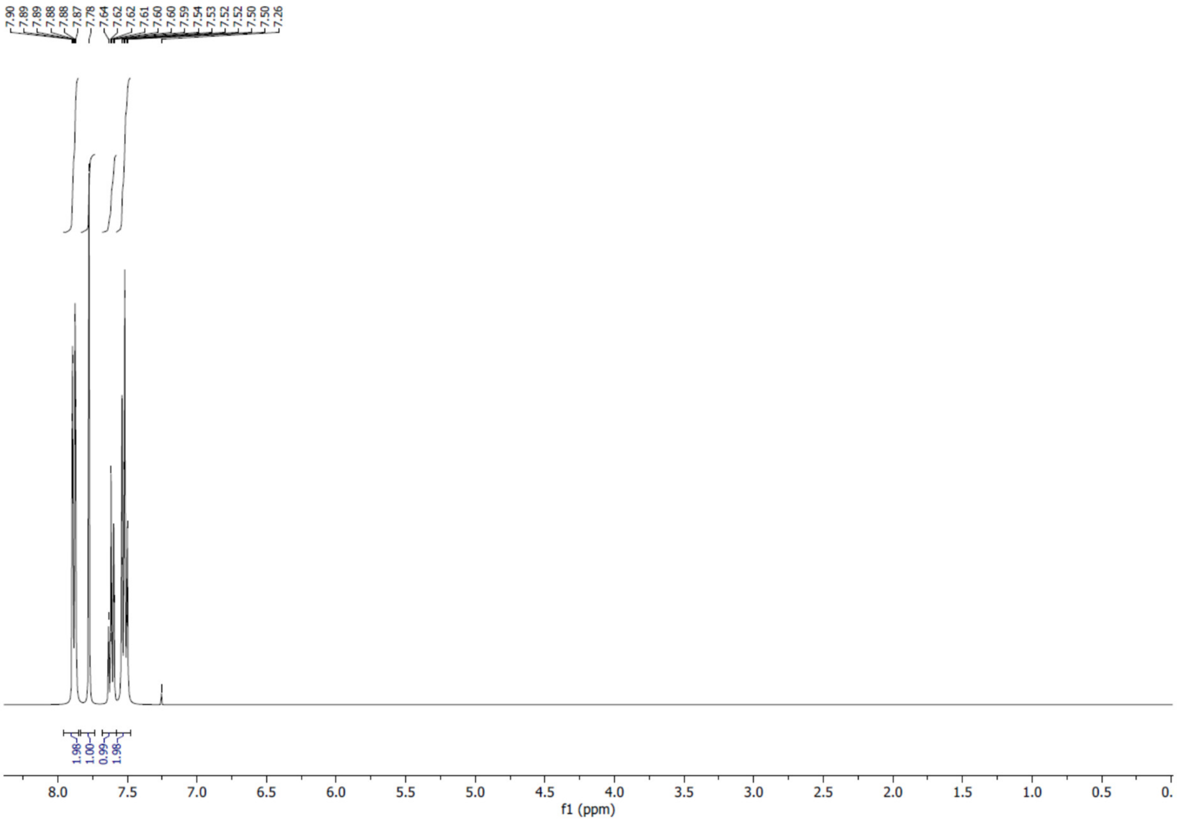

Figure S43. ^{1}H NMR (400 MHz, $CDCl_3$) spectra of benzylidenemalononitrile.

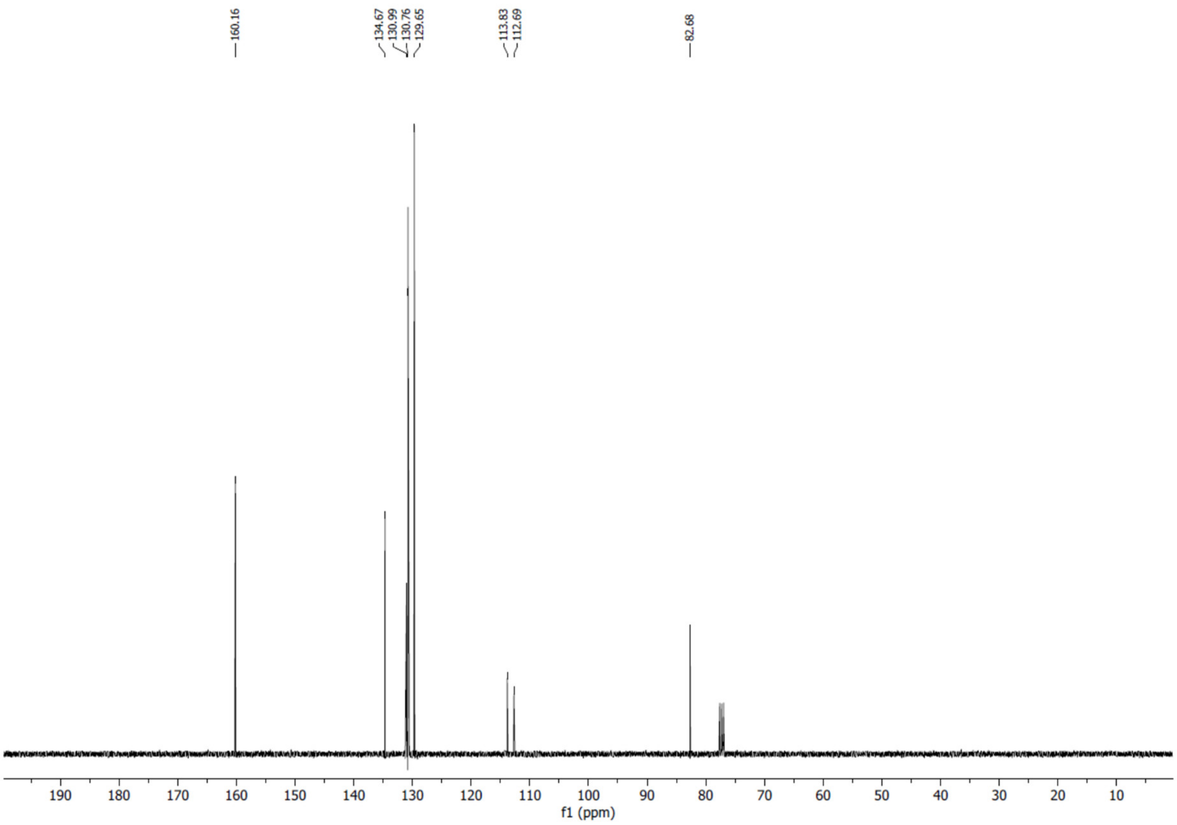

Figure S44. ^{13}C NMR (100 MHz, $CDCl_3$) spectra of benzylidenemalononitrile.
